# The Glycolytic Versatility of *Bacteroides uniformis* CECT 7771 and Its Genome Response to Oligo and Polysaccharides

**DOI:** 10.3389/fcimb.2017.00383

**Published:** 2017-08-25

**Authors:** Alfonso Benítez-Páez, Eva M. Gómez del Pulgar, Yolanda Sanz

**Affiliations:** Microbial Ecology, Nutrition & Health Research Unit, Institute of Agrochemistry and Food Technology (Instituto de Agroquimica y Tecnologia de Alimentos-Consejo Superior de Investigaciones Científicas) Valencia, Spain

**Keywords:** *Bacteroides uniformis*, polysaccharides, genome, transcriptome, mucin-degrader, GABA, butyrate

## Abstract

*Bacteroides* spp. are dominant components of the phylum Bacteroidetes in the gut microbiota and prosper in glycan enriched environments. However, knowledge of the machinery of specific species isolated from humans (like *Bacteroides uniformis*) contributing to the utilization of dietary and endogenous sources of glycans and their byproducts is limited. We have used the cutting-edge nanopore-based technology to sequence the genome of *B. uniformis* CECT 7771, a human symbiont with a proven pre-clinical efficacy on metabolic and immune dysfunctions in obesity animal models. We have also used massive sequencing approaches to distinguish the genome expression patterns in response to carbon sources of different complexity during growth. At genome-wide level, our analyses globally demonstrate that *B. uniformis* strains exhibit an expanded glycolytic capability when compared with other *Bacteroides* species. Moreover, by studying the growth and whole-genome expression of *B. uniformis* CECT 7771 in response to different carbon sources, we detected a differential growth fitness and expression patterns across the genome depending on the carbon source of the culture media. The dietary fibers used exerted different effects on *B. uniformis* CECT 7771 activating different molecular pathways and, therefore, allowing the production of different metabolite types with potential impact on gut health. The genome and transcriptome analysis of *B. uniformis* CECT 7771, in response to different carbon sources, shows its high versatility to utilize both dietary and endogenous glycans along with the production of potentially beneficial end products for both the bacterium and the host, pointing to a mechanistic basis of a mutualistic relationship.

## Introduction

The human gastrointestinal tract (GIT) is colonized by hundreds of microbial species exhibiting certain patterns of temporal succession and ecological niche (Koenig et al., 2011; Stearns et al., 2011). The number of studies concerning the inventory of microbiome functions and species inhabiting our GIT has reached a colossal magnitude, yielding valuable information of the bacterial genomes present in the human gut (Qin et al., 2010; Li et al., 2014). Diet is thought to be one of the main environmental factors modulating the gut microbiota to which gut microbes quickly respond (David et al., 2014). A vast amount of human studies indicate that plant-derived foods and especially dietary fiber beneficially impact bowel function and metabolic health; these effects could be partly mediated by gut microbiota (reviewed in Benitez-Paez et al., 2016a).

Most of the recent population human studies investigating the relationship between gut microbiota and lifestyle indicate that *Prevotella* spp., which belong to the Bacteroidetes phylum, is strongly associated with the intake of fiber-rich diets (De Filippo et al., 2010; Yatsunenko et al., 2012; Ou et al., 2013). Notwithstanding, *Bacteroides* spp., which are also dominant members of the phylum Bacteroides in humans, are also known to thrive in environments enriched in oligo and polysaccharides derived from plants (Matijasic et al., 2014). Indeed, a recent study demonstrated the oversimplification of associations established so far between the increased abundance of the genus *Prevotella* and consumption of plant-rich diets or that of *Bacteroides* and consumption of animal fat-protein-rich diets. This study indicated that sub-genus diversity should be taken into account, since different components of the genus *Bacteroides* could be associated to either plant-based or animal-based diets and the same applies to the genus *Prevotella* (De Filippis et al., 2016). The *sus* genes (from starch utilization system) of *Bacteroides* spp. have been characterized as the cornerstone for binding and importing of polysaccharide compounds (Reeves et al., 1997) and they are co-regulated with other genes encoding enzymes involved in glycan degradation in the so called PULs (polysaccharide utilization loci) (Martens et al., 2009).

*Bacteroides tethaiotaomicron* is considered as the archetype for studies of *sus* genes and PULs given the large number of genes encoded in its genome circumscribed to oligo and polysaccharide degradation function, a trait that confers to this species a high versatility to degrade a wide variety of glycan compounds (Xu et al., 2003). Moreover, the genome sequence of other *Bacteroides* species has permitted to expand the knowledge of the glycolytic potential of other species such as *B. cellulosyliticus* (McNulty et al., 2013). Likewise, reconstruction of genes for carbohydrate degradation from gut metagenomic data has indicated that *Bacteroides uniformis* also exhibits an important glycolytic capability (Tasse et al., 2010). Strikingly, the fibrolytic potential of *B. uniformis* was supported by a recent metagenomic study, which indicates that its dietary fiber degrading role is not restricted to the colon, but it extends to distal regions of the small intestine such as ileum (Patrascu et al., 2017).

A previous study investigating the colonization pattern of *Bacteroides* species in the full-term newborns' gut proposed that *B. uniformis* abundance is promoted by breast-feeding compared to formula feeding. (Sanchez et al., 2011). This finding suggests the potential of *B. uniformis* to thrive in the infant's gut from the early postnatal stage, presumably at expenses of the oligosaccharides present in the human milk (German et al., 2008). Our group has previously demonstrated *in vivo* the safety and effectiveness of *B. uniformis* CECT 7771 to ameliorate the metabolic and immune dysfunctions associated with obesity in mice (Gauffin Cano et al., 2012; Fernandez-Murga and Sanz, 2016). The objective of the present study was to uncover the potential glycolytic activity of *B. uniformis* CECT 7771 compared with other strains of the same species and to other *Bacteroides* species by an extensive comparative genome analysis. Secondly, we analyzed the genome response and metabolic output as a function of the carbon source available in the growth medium. To this end, we exposed *B. uniformis* CECT 7771 to a variety of oligo and polysaccharides in controlled anaerobic culture conditions and we also analyzed the bacterial cell response by a genome-wide exploratory gene expression analysis, quantitative PCR, and metabolite quantification analysis of end-products to identify the molecular pathways activated as a consequence of oligo- or polysaccharide fermentation.

## Methods

### Cells and culture media

*Bacteroides uniformis* CECT 7771 was isolated from stools of a healthy infant included in a prospective observational study carried out in a sub-group of 75 full-term newborns with at least one first-degree relative with celiac disease belonging to the Proficel study cohort (Sanchez et al., 2011). Infants were enrolled in the study after written informed consent was obtained from their parents as indicated elsewhere (Sanchez et al., 2011). *B. uniformis* CECT 7771 was grown for 48 h in Schaedler anaerobe agar plates (Oxoid) supplemented with 100 mg/L kanamycin, 7.5 mg/L vancomycin, and 0.5 mg/L vitamin K_3_. Bacteria were incubated at 37°C under anaerobic conditions into a Whitley DG250 Anaerobic Workstation (don Whitley Scientific, Inc., Shipley, UK). Single colonies were used to inoculate 10 mL of modified Schaedler broth (10 g/L tryptone soy broth, 2.43 g/L casein pancreatic peptone, 0.43 g/L soy peptone, 2.15 g/L meat extract, 5 g/L yeast extract, 5 g/L glucose, 0.75 g/L Tris-HCl, 0.4 g/L L-cysteine, 0.01 g/L hemin and 0.5 mg/L vitamin k_3_). The L-cysteine, hemin and vitamin K were filtered individually by using 0.22 μm disposable filters (Millipore) and then were added to the autoclaved media. Ten mL of an overnight culture of the strain studied was used for genomic DNA isolation. To evaluate the effects of different carbon sources on *B. uniformis* CECT 7771 growth, overnight cultures (0.7–0.8 OD_600_) of this bacterial strain were diluted 1/20 in fresh pre-warmed and oxygen-depleted modified Schadler media containing 0.5% w/v of the different carbon sources including glucose (Scharlau, Cat#GL01271000), inulin (Sigma, Cat#I2255), wheat bran extract (WBE) (Cargill, Antwerp, Belgium), gum arabic (Sigma, Cat#30888), pectin (Sigma, Cat#P9135), or type II mucin from porcine stomach (Sigma, Cat#M2378) and growth kinetics were monitored for 10 h. Each carbon source stock was sterilized by filtration using 0.22 μm disposable filters, thus avoiding the alteration of their original structure and chemical composition. The OD_600_ was measured at 1-h intervals for each sample until stationary phase. An aliquot of respective cultures was collected at exponential growth phase (OD_600_ ~0.4) for RNAseq analysis. The cells were then pelleted by centrifugation at 3,000 × g for 15 min at 4°C. The supernatant was aspired off, filtered using 0.22 μ filters and immediately stored at −80°C for further metabolite analyses and the cell pellet was stored at −80°C for RNA extraction. Three independent experimental trials were included in the analyses.

### DNA and RNA isolation

DNA and RNA from cell cultures were isolated using the MasterPure^TM^Gram Positive DNA Purification Kit (Epicenter) with slight variations over manufacturer's instructions. Briefly, a cell lysis step was improved by incubating each cell suspension with 500 mg Lysozyme (Sigma, Cat #62970) and 20 U Mutanolysin (Sigma, Cat #9901) for 60 min at 37°C. For RNA isolation samples were incubated with 2 U DNase I (Epicenter) at 37°C for 60 min instead of the RNase A treatment.

### Genomic DNA sequencing

*B. uniformis* CECT 7771 DNA was sequenced using MinION portable DNA sequencer and flowcells based on the R7.3 and R9.4 pore chemistries. The R7.3 run consisted of a DNA library prepared with the Genomic DNA Sequencing Kit SQK-MAP006 (Oxford Nanopore Technologies) and 6 μg genomic DNA according to manufacturer's instructions. The genomic DNA was sheared with gTUBE^TM^ (Covaris) to get fragments of about 10 Kbp, the sheared DNA was then repaired with the PreCR® Repair Mix (New England Biolabs) followed by purification using Agencourt AMPure XP beads (Beckman Coulter). DNA attached to AMPure beads was washed twice with freshly prepared 70% ethanol and magnetic rack. The DNA was eluted in 46 μL nuclease-free water and quantified by using Qubit 3.0 fluorometer and the Qubit dsDNA HS Assay Kit (Thermo Fisher Scientific). One microgram of repaired DNA was further processed using the NEBNext Ultra II End Repair/dA-Tailing Module (New England Biolabs) followed by a new round of washing in the magnetic rack as previously done. The DNA was eluted from magnetic beads using 38 μL nuclease-free water and adapter ligation step was conducted by adding 10 uL Adapter Mix, 2 uL HP adapter, and 50 uL Blunt/TA Master Mix (New England Biolabs) mixing by inversion between each sequential addition. After 15 min incubation at room temperature 1 μL HP Tether was added to ligation reaction and incubation was extended for 10 min. The adapter-ligated genomic DNA was recovered with Dynabeads® MyOne Streptavidin C1 beads (Thermo Fisher Scientific) a magnetic rack, and Elution Buffer (SQK-MAP006 kit). Approximately, 205 ng DNA library were recovered and they were loaded into a brand new, sealed R7.3 flowcell previously fitted to the MinION™and primed twice with 71 μL premixed nuclease free water, 75 μL 2x running buffer, and 4 μL fuel mix. The initial sequencing mix was prepared with 56 μL nuclease-free water, 75 μL 2X running buffer, 4 μL fuel mix, and 15 μL DNA library (~120 ng). A standard 48-h sequencing protocol was initiated using MinKNOW^TM^ v0.50.2.15. The base-calling was performed through data transference using the Metrichor™ agent v2.36.2 and 2D Basecalling workflow v1.62. During the sequencing run, the remained aliquot of DNA library was loaded in equally conditions after 24 h of initial input. The R9.4 run consisted of a DNA library prepared with the 1D Genomic DNA Sequencing Kit SQK-LSK108 (Oxford Nanopore Technologies) and 3 μg genomic DNA according to manufacturer's instructions. Briefly, the DNA repair kit used was NEBNext FFPE RepairMix (New England Biolabs) and the final DNA library purification was performed with AMPure beads instead Dynabeads®. The sequencing mix was obtained by combining 37.5 μL running buffer, 25.5 μL LLB beads, and 12 μL DNA library (~240 ng) and loaded into the spot-on port of R9.4 flowcell previously mounted into a MinION^TM^ MkIb sequencer. A standard 48-h sequencing protocol was initiated using MinKNOW^TM^ v1.1.17. The base-calling was performed through data transference using the Metrichor™agent v2.43.1 and 1D BasecallingFLO-MIN106 450bs workflow v1.121. During the sequencing run, the remained aliquot of DNA library was loaded in equally conditions after 12 h of initial input. This sequencing run was extended for 24 h in sum.

### RNA sequencing

The total RNA obtained was quantified by using the Qubit 3.0 fluorometer and Qubit® dsDNA HS Assay Kit (Thermo Fisher Scientific). Thus, 30 μg total RNA were obtained by pooling respective replicates of different culture conditions in equimolar quantities (10 μg per replicate). RNA pools were sent to Eurofins Genomics GmbH (Ebersberg, Germany) to produce cDNA libraries with an insert size of ~400 bp with prior rRNA depletion using RiboZeroTM Magnetic Kit Gram-Negative Bacteria (Epicenter). The six cDNA libraries (glucose, WBE, pectin, inulin, gum arabic, and mucin) were pooled and sequenced in one Illumina HiSeq2500 channel with chemistry v4 and configuration 2 × 125 paired-end reads.

### Data analysis

Quality assessment of fast5 files and conversion to fastq and fasta formats was performed using the poRe (Watson et al., 2014) package v0.17. The *B. uniformis* CECT 7771 genome assembly was performed with 2D reads from R7.3 chemistry (32,764 reads), the high-quality set of 1D reads from R9.4 chemistry (10,598 reads), and the Canu assembler v1.3 (Koren et al., 2017) with options *genomeSize* = 4.5 m, *minReadLength* = 500, *corMinCoverage* = 5. A genome sequence refinement was performed with the RNAseq data derived from Illumina HiSeq2500 consisting of ~275 million paired-end reads, which were mapped against the draft genome of *B. uniformis* CECT 7771 using *bowtie2* (Langmead and Salzberg, 2012) for aligning and *samtools* v1.3.1 (Li et al., 2009) set of algorithms for indexing, sorting, and pileup of mapped reads. A consensus sequence was recovered and further processed to allow super-scaffolding. This last step was completed by performing *de novo* transcriptome assembly using the entire set of RNAseq paired-end reads and the *velvet* v1.2.10 and *oases* v0.2.09 assemblers using 63 as k-mer parameter (Zerbino and Birney, 2008; Schulz et al., 2012). Thus, the entire set of contigs assembled (31,576 in total) were mapped against the draft genome of *B. uniformis* CECT 7771 obtaining a new and more refined consensus sequence. Finally, a blast-based comparison among refined scaffolds of *B. uniformis* CECT 7771 was carried out to detect strong similarity among edges. This information was used to perform manual assembly of the 11 initial scaffolds generated from Canu into 6 superscaffolds. The genome assembly of the *B. uniformis* CECT 7771 was submitted to the European Nucleotide Archive (ENA) where it is publicly available under primary accession number PRJEB19372 (ENA WGS accessions: FZQS01000001-FZQS01000006). Comparative genomics against the *B. uniformis* ATCC 8492 (ENA WGS accession: AAYH02000000), *B. uniformis* CL03T00C23 (ENA WGS accession: AGXY01000000), *B. uniformis* dnLKV2 (ENA WGS accession: ASSO01000000), and *B. uniformis* 3978-T3i (ENA WGS accession: JNHO01000000) was assessed with alignment algorithms implemented in Mauve v2.3.1 (Darling et al., 2010) and BRIG (Alikhan et al., 2011). Furthermore, genome-wide average nucleotide identity approach implemented in JSpecies v1.2.1 (Richter and Rossello-Mora, 2009) was used to reconstruct phylogenetic relationships among *B. uniformis* species and other *Bacteroides* such as *Bacteroides vulgatus* ATCC 8482 (ENA accession: CP000139.1), *Bacteroides fragilis* NCTC 9343 (ENA accession: CR626927.1), *Bacteroides thetaiotaomicron* VPI 5482 (ENA accession: AE015928.1), *Bacteroides caccae* ATCC 43185 (ENA WGS accession: AAVM02000000), and *Bacteroides stercoris* ATCC 43183 (ENA WGS accession: ABFZ02000000). Values from ANIm and tetranucleotide distribution were used to reconstruct a UPGMA dendrogram (http://genomes.urv.cat/UPGMA) using the RSMD distance coefficient and the iTOL web server (Letunic and Bork, 2016) was employed to draw the respective rooted trees. Gene prediction and functional annotation were achieved by using Prodigal v2.6 (Hyatt et al., 2010), tRNAscan v1.4 (Lowe and Eddy, 1997), RNAmmer v1.2 (Lagesen et al., 2007), CRISPR Recognition Tool (CRT) (Bland et al., 2007), KEGG Automatic Annotation system (Moriya et al., 2007), CAZy database (Lombard et al., 2014), SMART database (Letunic et al., 2014), and CAT server (Park et al., 2010). Venn diagrams were designed in *jvenn* server (Bardou et al., 2014). Assessment for presence of virulence factors, antibiotic resistance genes, and toxins was done by executing local blast to compare all the ORFs predicted against the annotated proteins present in the VFDB (Chen et al., 2016), MvirDB (Zhou et al., 2007), and ARDB (Liu and Pop, 2009) databases. Processing of RNAseq data was done according to previous studies (Benitez-Paez et al., 2016b). Briefly, the quality filtering and trimming were performed using FASTX-toolkit (http://hannonlab.cshl.edu/fastx_toolkit/). Read mapping was assisted by Blast algorithm (Altschul et al., 1990) and selecting alignments >50% of read length (>70 nt) and 100% identity. Read counts were normalized using RPKM and a genome-wide exploratory differential expression among transcriptomes generated from usage of different carbon sources was measured with GFOLD (Feng et al., 2012) using the glucose-derived expression as baseline feature and followed by further evaluation with qPCR methods. In order to increase the stringency for detecting more probable signals of differential expression, we only selected genes with GFOLD score ≤ −1.5 or ≥1.5 (log_2_ fold-change) despite than any gene with GFOLD score different than zero would be indicative of up- or down-regulation. Sequence information supporting the six *B. uniformis* CECT 7771 transcriptome analyses was submitted to the ENA where it is publicly available under primary accession number PRJEB19372. Hierarchical clustering of genes differentially expressed in different culture conditions was achieved by using Euclidean distance and average linkage methods (Metsalu and Vilo, 2015).

### Quantitative PCR

The genes BUNIF7771_0387, BUNIF7771_0544, BUNIF7771_0548, BUNIF7771_1668, BUNIF7771_1883, BUNIF7771_3473, BUNIF7771_3732, and BUNIF7771_4131 were selected from the exploratory RNAseq analysis to assess specific changes in expression by qPCR. The gene-specific oligonucleotides used for this aim are presented in the Table S1. The cDNA was synthesized using 5 μg of total and non-pooled RNA remaining from that used for the RNAseq approach (three replicates per treatment), and the High Capacity cDNA Reverse Transcription Kit (Applied Biosystems) according to the manufacturer's instructions. The qPCR reactions were set in 96-well plates using the SYBR Green I Master Mix (Roche Lifesciences), 0.5 μM of forward oligonucleotide, 0.25 μM of reverse oligonucleotide, and 1 μL of the cDNA reaction. All treatment samples were set in triplicate in the plate and amplified in a LightCycler 480 II with the following cycling profile: initial incubation at 95° for 5 min and 40 cycles of 10 s at 95°, 20 s at 63°, and 15 s at 72°. Finally, the melting curve was set from 65 to 97° with a ramp rate of 0.11°/s. The expression level for each gene was measure according to the ΔΔCt method, using the expression of the 16S rRNA gene as calibrator, and expression of glucose samples as reference. RQ values were finally obtained with calculation of 2^−ΔΔCt^ for all samples and replicates. Differential expression was assessed by the one-sided *t*-test with Welch's correction supporting pairwise comparisons between gene expression under glucose and remaining treatments. The copy number of the pBU7771 extrachromosomal element was calculated by absolute quantification using the primers pBU7771-F and pBU7771-R (Table S1). The single-stranded DNA (ssDNA), fully covering the region to be amplified (111 nt) was obtained from Isogen Life Science B.V (Utrecht, The Netherlands) where it was synthesized, PAGE-purified, and quantified, and used in molecule titration during qPCR. The number of plasmid molecules per ng DNA was obtained and divided by the theoretical number of *B. uniformis* CECT 7771 genomes presented in 1 ng DNA (5.16 Mb) (http://cels.uri.edu/gsc/cndna.html), this ratio was used to infer the number of plasmids per cell.

### Metabolite quantification

The gamma-amino butyric acid (GABA) concentration was analyzed in supernatants (150 μL) obtained from cultures supplemented with either glucose, mucin or pectin and collected at OD_600_ ~ 0.4 by LC/MS approach in the Central Service for Experimental Research (SCSIE) at the University of Valencia. The GABA (Sigma, Cat#A2129) standards were prepared in milliQ grade water. Both the standards and samples were diluted 1:4 with acetonitrile in 200 μL reaction volume, then samples were centrifuged at 10,000 × g for 10 min to facilitate protein precipitation and 50 μL of supernatant were loaded in the ACQUITY® TQD (Waters Corporation. Milford, MS, USA) LC instrument coupled to an electrospray ionization (ESI). GABA was separated in a HILIC Kinetex column (Phenomenex®) using 0.1% formic acid: 75% acetonitrile at 23°C. The area under the curve (AUC) of GABA standards at 1, 0.1, 0.01, and 0.001 mM were used to quantify its concentration in the culture supernatants. Statistical analysis was done with the values of three independent assessments using one-way ANOVA with pairwise comparisons and Bonferroni correction.

## Results

### The genome of *Bacteroides uniformis* CECT 7771

Following an extensive sequence analysis of the genomic information retrieved from a third generation sequencing platform, the MinION^TM^, we could initially assemble the *B. uniformis* CECT 7771 genome which consisted of ~5.16 Mbp with a N50 of 1.32 Mbp arranged in 11 scaffolds. The MinION^TM^ data is characterized by producing error-prone DNA reads with a per-base accuracy above 85% in 2D reads (Ip et al., 2015), therefore we assumed further and hybrid approaches to get genome sequence refinement. After refinement steps with RNAseq data, using individual reads and assembled into longer transcript contigs, derived from six different experiments to assess the transcriptional profile under different carbon sources, we obtained a high quality genome assembly consisting of 5.16 Mpb with a N50 2.44 Mpb and arranged in 5 super-scaffolds representing the chromosomal genetic information of *B. uniformis* CECT 7771. This genome information was submitted to the European Nucleotide Archive (ENA) and can be accessed with identifiers FZQS01000001 to FZQS01000005. Additionally, we found an extra-chromosomal element of 2,746 bp in length that seems to encode neither virulence nor antibiotic resistance genes (ENA accession FZQS01000006). By absolute quantification approaches, we found the pBU7771 plasmid is present in very-low copy number reaching a proportion of 1.86 plasmids per cell. We have predicted that in the current version of the *B. uniformis* CECT 7771 genome there are 5,226 ORFs, 4 ribosomal RNA operons, and 67 tRNAs encoded. Moreover, we found one CRISPR region characterized to have 11 repeats consisting of the following DNA sequence: GTTGTGATTTGCTTTCATTTTAGTATCTTTGAACCATTGGAAACAGC and 10 spacers with an average length of 30 nt. This last feature was confirmed by the fact that the downstream of this CRISPR region are contiguously located the BUNIF7771_0521 and BUNIF7771_0522 ORFs encoding the Cas2 and Cas1 CRISPR-associated endonucleases, respectively. Ribosomal RNA sequence information of *B. uniformis* CECT 7771 is publicly available in the ENA under accession numbers LT745888, LT745889, and LT745890 for 5S, 16S, and 23S molecules, respectively. An initial comparison with other genome assemblies from *B. uniformis* strains is presented in Table 1. The genome assembly presented in this study fully fits the features observed for previously sequenced *B. uniformis* strains. Notably, the draft genome of *B. uniformis* CECT 7771 share similar GC content with its counterparts being 46.7% and constitute the largest genome assembly of any *B. uniformis* published with the lowest number of scaffolds reconstructed. In this regard, it seems that the hybrid assembly strategy based on long read sequencing approach followed by refinement with massive short read data resulted in a valid methodology to sequencing the *B. uniformis* CECT 7771 genome retrieving more sequence information to distinguish interesting features of *B. uniformis* strains. Among such features, we highlighted the presence of 4 complete ribosomal RNA operons whose number was not consensual from previous assemblies. Besides, we reported for the first time the presence of a plasmid element in a *B. uniformis* strain of 2,746 bp in length. Using this element as query in a Blast searching against the NCBI's non-redundant nucleotide database we observed that this element was commonly found in other *Bacteroides* species consisting of an extrachromosomal DNA element that was also recovered from metamobilome studies in rat cecum (Jorgensen et al., 2014) with a high sequence similarity to a plasmid element borne by *B. fragilis* IB143 (Smith et al., 1995). Notwithstanding, none of the three ORFs predicted to be encoded by this element match with any antibiotic resistance, virulence, or pathogenic gene annotated in the ARDB, MvirDB, and VFDB databases.

**Table 1:** Genome assembly comparison among different strains of *B. uniformis*.

**Feature**	***B. uniformis* CECT 7771**	***B. uniformis* ATCC 8492**	***B. uniformis* CL03T003C23**	***B. uniformis* 3978-T3i**	***B. uniformis* dnLKV2**
Sequencing platform	MinION^TM^ HiSeq2500	454 GS20	HiSeq2000	HiSeq2500	HiSeq2000
Assembly length	5.16 Mbp	4.72 Mbp	4.96 Mbp	5.05 Mbp	4.84 Mpb
Scaffolds	6	33	8	47	11
N50	2.44 Mbp	0.29 Mbp	4.92 Mbp	1.01 Mbp	0.95 Mbp
Plasmids	1	0	0	0	0
RNA ribosomal operons^a^	4	4^b^	4	6	3
GC%	46.7	46.4	45.9	46.4.	46.3

*The assembly statistics for B. uniformis ATCC 8492, CL03T003C23, 3978-T3i, and dnLKV2 strains were recovered from information available at ENA for respective assembly accessions (see Methods)*.

a *Number of RNA operons estimated through analysis of respective assemblies with RNAmmer v1.2*.

b *Calculated from the maximum number of 16S and 5S rRNA genes found given that only one 23S rRNA gene was found*.

**Figure 1:**
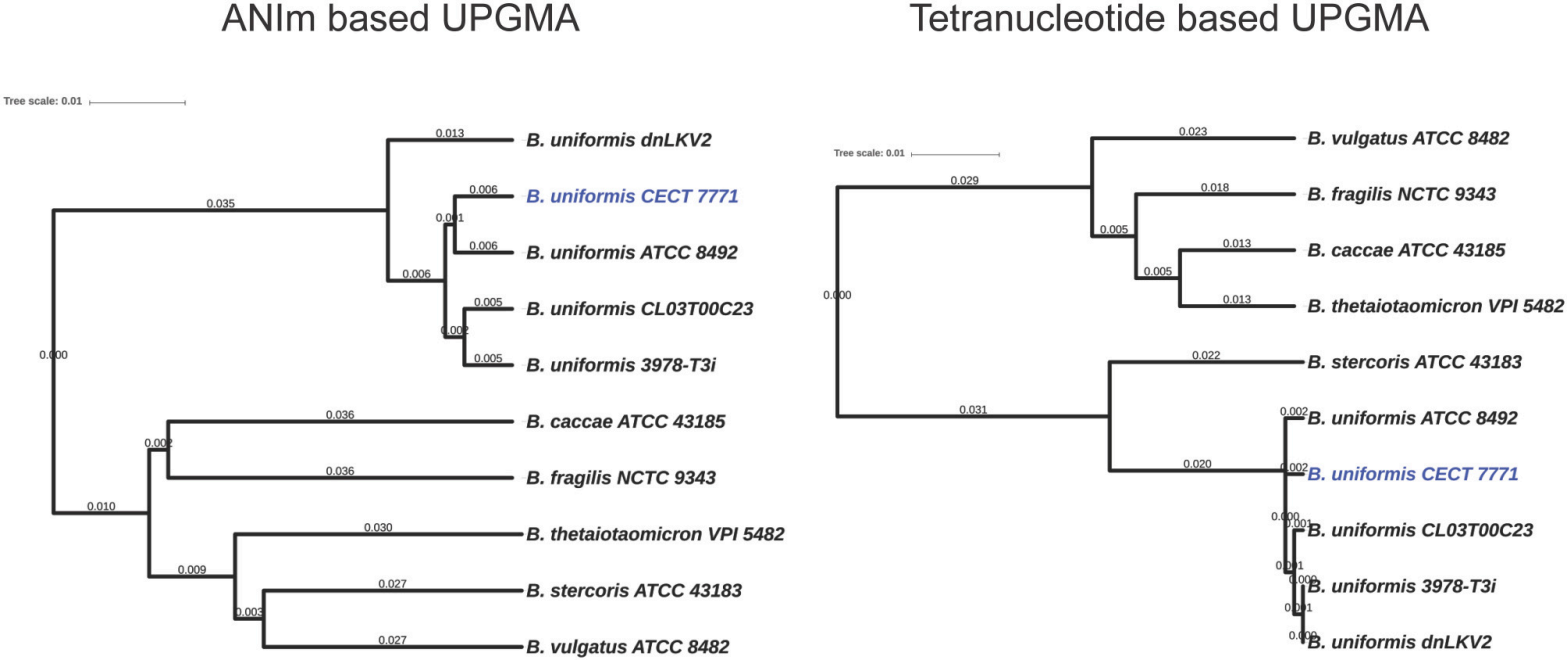
Phylogenetic relationships among *Bacteroides* species. The genome information of all species and strains presented in the figure was used for comparative analysis using algorithms implemented in the Jspecies tool (Richter and Rossello-Mora, 2009). Genetic distance based on the Average Nucleotide Identity is presented in the left UPGMA dendrogram. The relationships based on tetranucleotide distribution is presented in the UPGMA right dendrogram. Branch lengths are based on the RSMD distance coefficient.

**Figure 2:**
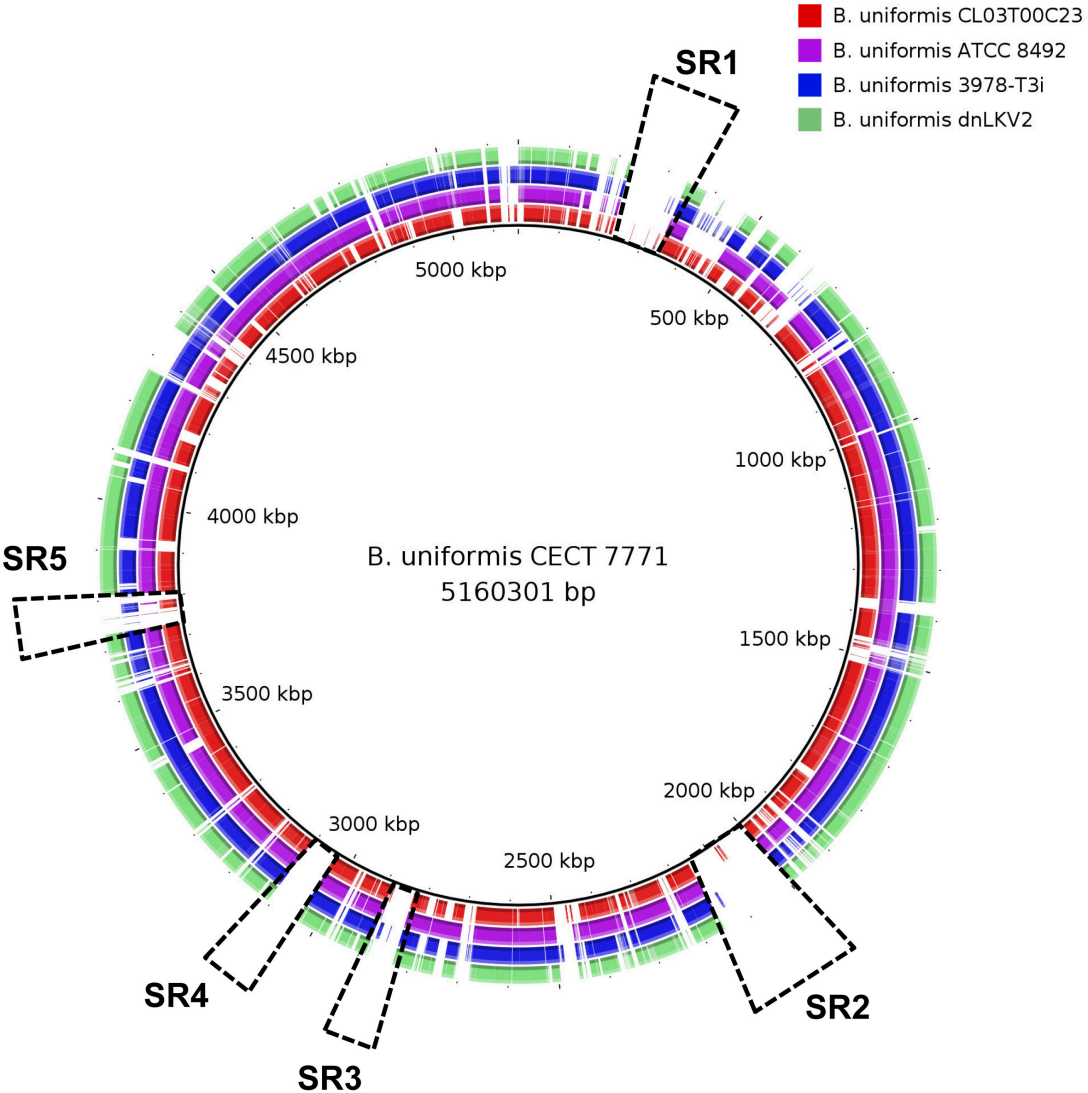
Comparative analysis of *Bacteroides uniformis* strains. Circular representation of *B. uniformis* CECT 7771 (black inner line), *B. unformis* CL03T00C23, (red), *B. unformis* ATCC 8495 (purple), *B. unformis* 3978-T3i (blue), and *B. unformis* dnLKV2 (green) genomes. They are compared using whole-genome and blast-based alignment. Genomic regions exclusively found in *B. uniformis* CECT 7771 are highlighted with dashed rectangles.

Comparative genomics approaches based on whole-genome alignment were used to identify distinctive features of *B. uniformis* CECT 7771 compared to other strains belonging to the same and to other species. The average nucleotide identity and tetranucleotide distribution across the genome indicated that *B. uniformis* CECT appears to be very related to other *B. uniformis* strains but its genomic structure also differs from that of other *Bacteroides* species (Figure 1 and Table S2). A more detailed comparison among different *B. uniformis* strains has led to identify genomic regions exclusively present in the genome of *B. uniformis* CECT 7771, named specificity regions hereinafter (SR1–SR5, Figure 2). Despite the low level of functional annotation recovered when ORFs encoded into the major SR regions were analyzed by KAAS server (Moriya et al., 2007), we could identify interesting features such as presence of endopeptidases (SR1 and SR3), DNA methylases (SR1, SR2, and SR3), a type-IV restriction endonuclease (SR3), a two-component system (SR1), and polysaccharide metabolism associated enzymes (SR2) for importing, degradation and synthesis.

**Figure 3:**
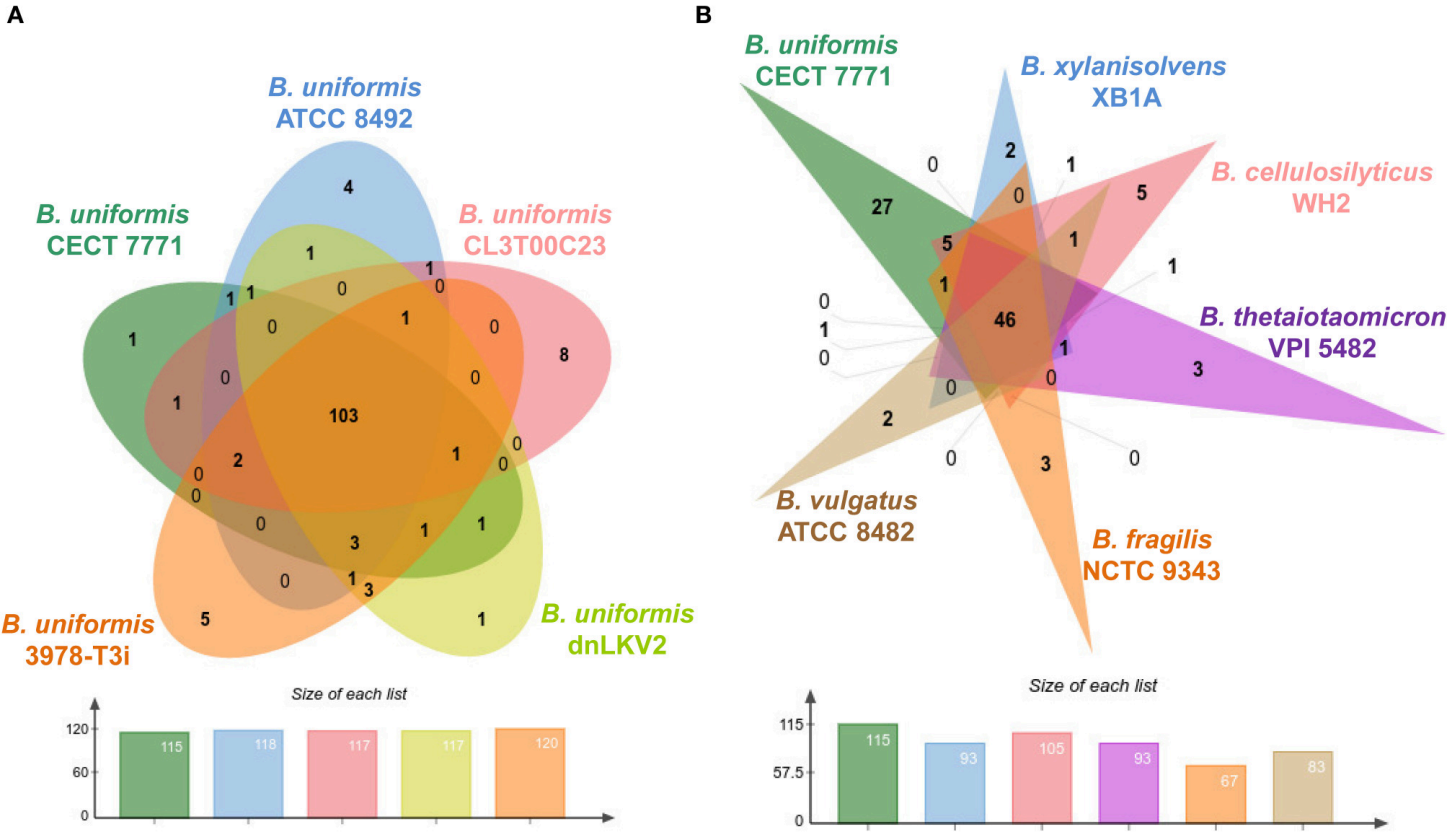
*B. uniformis* CECT 7771 and carbohydrate metabolism. **(A)** Venn diagram showing the strain-specific and shared CAZy families among five *B. uniformis* strains. **(B)** Venn diagram showing the species-specific and shared CAZy families among six *Bacteroides* species. The number of non-redundant CAZy families present in respective genomes is showed below respective Venn diagrams following the color nomenclature.

The members of the phylum Bacteroidetes are well known components of the gut microbiota thriving in environments enriched in glycans. The *sus* genes, an archetypal locus of membrane proteins for binding and importing polysaccharides in Bacteroidetes species, are a fundamental part of the PULs. The number of these loci specialized in glycan degradation in *Bacteroides* spp. and *Prevotella* spp. species outnumbers the number of PULs found in species of other phyla such as Firmicutes (White et al., 2014). Therefore, we wanted to survey the number and classes of enzymes involved in binding, degradation, modification, and synthesis of glycans in the *B. uniformis* CECT 7771 genome. Using the CAT server (Park et al., 2010), we retrieved annotation of ORFs encoding enzymes homologs to reference genes present in the CAZy database (Lombard et al., 2014). A first comparison was accomplished at strain level using the genome information from *B. uniformis* strains listed in Table 1. Consequently, we have recovered a total of 622, 626, 605, 624, and 671 CAZy genes for *B. uniformis* CECT 7771, *B. uniformis* ATCC 8492, *B. uniformis* CL03T00C23, *B uniformis* dnLKV2, and *B. uniformis* 3978-T3i, respectively. Those genes were grouped into non-redundat CAZy family domains, then families were split in case of multi-domain proteins and finally compared via Venn diagrams to disclose function uniqueness in every single strain (Figure 3A). We found a similar number of families in all strains and the assembled *B. uniformis* CECT 7771 genome encodes 115 different CAZy functional families whereas other strains encode up to 120. Interestingly, the five strains share in average 88% (103/117) of CAZy families and up to 90% (122/135) in the case of pairwise comparisons. When strain-specific functions were explored, we found that all strains showed unique features encoded in their respective chromosomes (Table S3). Although the glycosidehydrolase (GH) function was predominantly recovered in a set of unique genes in each strain, the presence of glycosyl transferase (GT) activities was only observed in *B. uniformis* CECT 7771 and 3978-T3i strains. The GT catalyzes the transference of sugar moieties from an activated donor to a specific substrate in an enzyme-dependent manner. In addition to the GT genes exclusively present in the genomes of strains of *B. uniformis*, the strains the *B. uniformis* CECT 7771 and 3978-T3i strains exhibited the largest number of GT enzyme encoded genes (30) when compared with counterparts (25 genes per strain on average). Therefore, the gain of GT genes would be indicative of the higher capability of these strains to synthesizing disaccharides, oligosaccharides, polysaccharides, and even glycolipids. Accordingly, *B. uniformis* CECT 7771 could represent an important and unexplored reservoir of GT functions for production of natural compounds with an ample range of biotechnology applications.

On the other hand, comparisons at species level were done using similar analysis as the previous one but with the genomic information of the annotated genes in the CAZy database and available for *Bacteroides* species such as: *B*. *xylanisolvens* XB1A, *B. cellulosilyticus* WH2, *B. thetaiotaomicron* VPI5482, *B. vulgatus* ATCC 8482 and *B. fragilis* NCTC 9343. Strikingly, the number of CAZy genes encoded in other *Bacteroides* species is lower than that detected in *B. uniformis* strains (Figure 3B). The above observation reveals a new degree of specialization of *B. uniformis* strains that outnumber the CAZy genes found in other *Bacteroides* species even in *B. thetaiotaomicron*, the archetype for characterization of PULs and *B. cellulosilyticus* previously described to encode an expanded glycolytic potential (McNulty et al., 2013). In the same line of thinking, almost the 23% (27/115) of *B. uniformis* CECT 7771 CAZy genes cannot be identified in other species. Moreover, the number of CAZy genes present in *Bacteroides* spp. genomes seems to be indicative of their role as commensals or potential pathogens. Thus, in species like *B. vulgatus* and *B. fragilis*, which are considered as pathobionts (Wexler, 2007) the number of these genes are abnormally lower than in other species; this is particularly notable for *B. fragilis* which has almost half of genes found in *B. uniformis* strains.

Finally, a searching for potential antibiotic resistance and virulence genes was completed by comparing the full set of ORFs predicted to be encoded in the *B. uniformis* CECT 7771 genome according to the proteins annotated in the ARDB, MvirDB, and VFDB databases. We detected presence of a β-lactamase protein encoded in the ORF BUNIF7771_3570 (99% sequence identity against the AAA66962 ARDB entry) and presence of the TetQ protein encoded in the ORF BUNIF7771_4507 (90% sequence identity against the AAS83507 ARDB entry), both proteins with homologs in other *B. uniformis* genomes analyzed in above sections. An additional KEGG based functional analysis of ORFs predicted to be encoded by the *B. uniformis* CECT 7771 genome has revealed the presence of a total of 24 genes corresponding to 11 different multidrug efflux pumps, one D-alanyl-D-alanine dipeptidase (*vanX*, K08641) associated with vancomycin resistance, and a couple of genes associated with cationic antimicrobial peptide resistance.

### *B. uniformis* CECT 7771 growth fitness depending on the carbon source utilization

**Figure 4:**
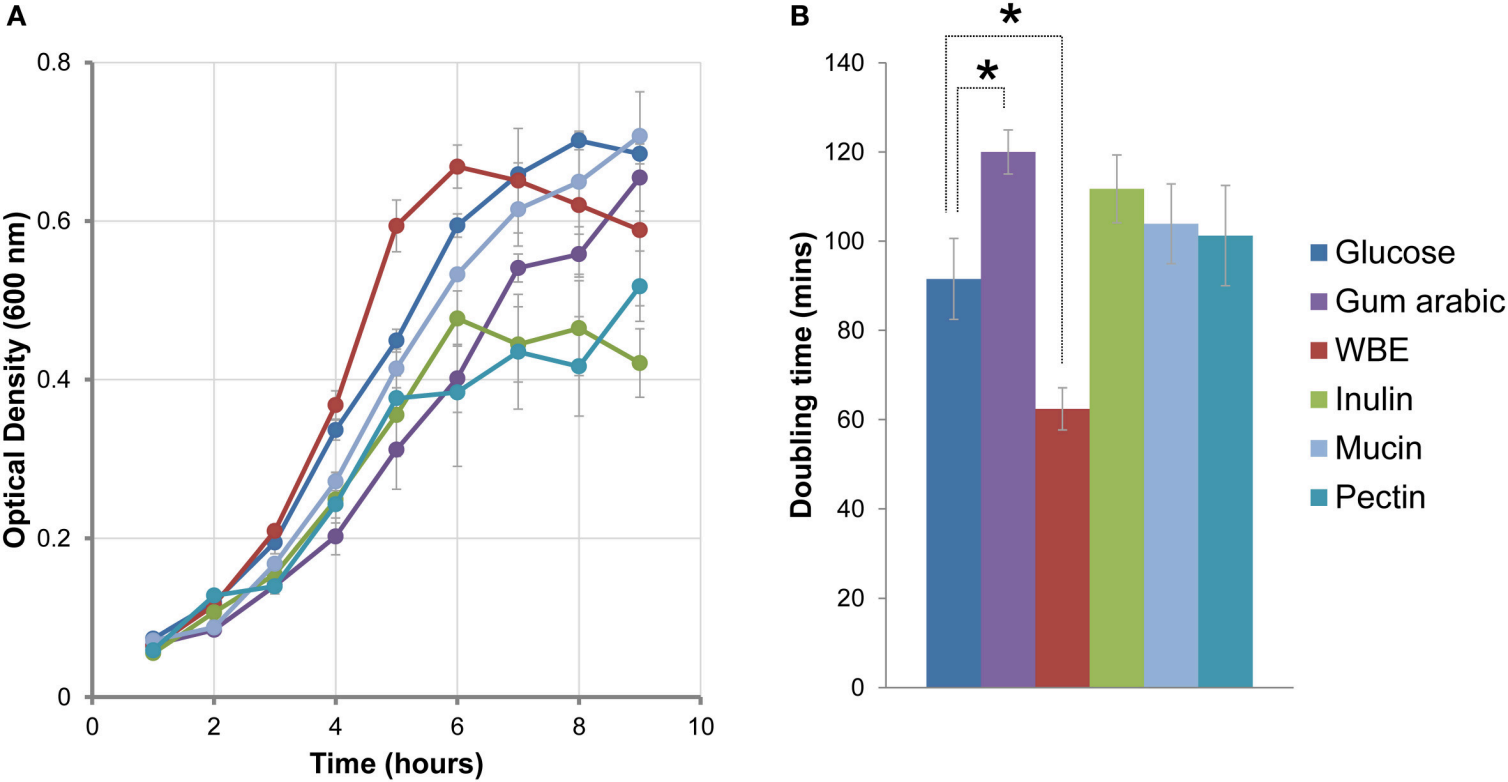
Growth fitness of *B. uniformis* CECTC 7771 in different carbon sources. **(A)** Growth curve comparison between *B. uniformis* CECT 7771 cultures using glucose (blue line), gum arabic (purple), WBE (red), inulin (green), pectin (baby blue), or mucin (blue-green) as the carbon source. Growth was monitored measuring OD_600_ at 60 min intervals. The OD_600_ values are presented as a mean of three independent replicates (±SEM). **(B)** Doubling times calculated during the exponential growth phase by linear regression. The values are present as a mean of three independent replicates (±SEM). The asterisks indicate differential growth rates when compared to glucose used as the reference (*p* < 0.022 for gum arabic and *p* < 0.057 for WBE) supported on pairwise *t*-test comparison with Welch's correction.

In the light of the wide repertoire of genes dedicated to polysaccharide degradation identified in *B. uniformis* CECT 7771 genome, we wanted to investigate those expressed in response to complex carbon sources that can be present in the intestinal tract (GIT) such as mucin, a heavily glycosylated secretion product of the intestine epithelial cells. To do so, the ability of *B. uniformis* CECT 7771 to grow in the presence of glucose, gum arabic, WBE, inulin, mucin, or pectin as primary carbon source was evaluated. Growth patterns differed (Figure 4A) essentially regarding the log phase and the yield at stationary phase. To quantify the ability to growth in the different carbon sources we also calculated the doubling time during the exponential growth phase (Figure 4B). In general, we observed that all carbon sources except for WBE increased the doubling time of the bacterium compared to glucose; this effect was especially significant for gum arabic (*p* < 0.022). Notably, WBE showed no fitness cost and boosted the *B. uniformis* CECT 7771 growth reducing the doubling time from 91 to 62 min (*p* < 0.057). The opposite effects of gum arabic and WBE on growth fitness could be explained by their composition. Whereas gum arabic consist of a complex mixture of branched polymers of galactose (39–42%), rhamnose (12–16%), arabinose (24–27%), and glucuronic acid (15–16%) (Ali et al., 2009), the WBE is principally composed of AXOS (>69%), which are a mixture of low molecular weight xylo- and arabinoxylo-oligosaccharides (Cargill, Antwerp, Belgium). The complex polysaccharides contained in gum arabic, inulin, and pectin seem to be fermented with more difficulty by *B. uniformis* CECT 7771 probably by the lacking of the proper de-branching enzymes useful in the first hydrolytic stages from poly- to oligosaccharides. Therefore, *B. uniformis* CECT 7771 is more specialized in the utilization of oligosaccharides than in very complex carbohydrates, which could also be related to the strain origin (isolated from stools of a breast-fed infant).

### Genome response of *B. uniformis* CECT 7771 to different carbon sources

**Figure 5:**
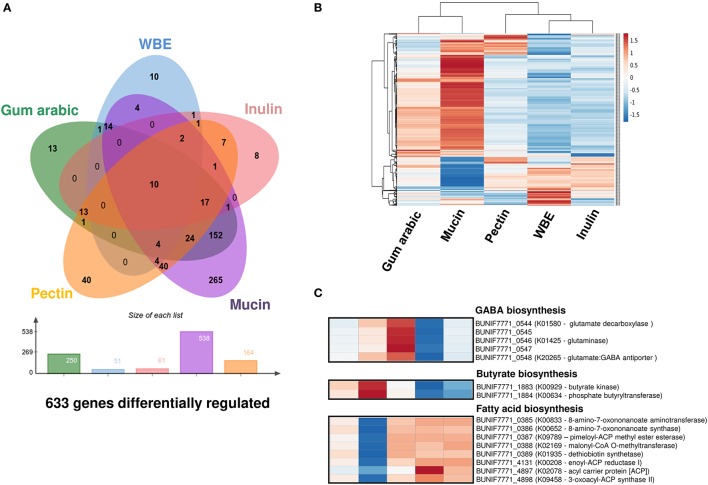
Expression patterns in the *B. uniformis* CECT 7771 genome. **(A)** Venn diagram showing the number of genes differentially expressed in *B. uniformis* CECT 7771 cultures using gum arabic, WBE, inulin, pectin, and mucin as the carbon source. The expression pattern in glucose media was used as normalizer. The total number of genes showing differential expression in different conditions are depicted below the Venn diagram. **(B)** Heatmap with expression values obtained from the exploratory transcriptome analysis of the 633 genes showing differential expression in all condition tested. Hierarchical clustering was assessed for the treatments and genes using Euclidean distance and average linkage methods implemented in ClustVistweb server. **(C)** Detailed view of the gene expression profile for 15 genes associated with GABA, butyrate, and long-chain fatty acid biosynthesis. Both the treatment labels and gene expression score correspond with those observed in **(B)**.

With the aim to complement the study above, we also did an exploratory genome-wide expression analysis in the presence of gum arabic, WBE, inulin, mucin, and pectin. This analysis was done using a RNAseq approach with RNA pools from respective replicates obtained in different and independent experiments. Using comparative expression algorithms implemented in GFOLD (Feng et al., 2012) we could discern a set of genes more probable to be differentially regulated under the respective conditions. By setting an increased threshold for selection (see Methods) we obtained a total 633 genes with a high probability to be differentially expressed among all conditions (Figure 5A). Interestingly, the gum arabic and mucin seem to induce and attenuate the expression of a larger set of genes in *B. uniformis* CECT 7771. Moreover, both carbon sources similarly modify the expression of 152 out of 633 (24%) genes differentially expressed in all conditions inducing a similar response. This is consistent with what graphically retrieved when a hierarchical clustering approach was used to study the particular gene expression patterns for the 633 genes with plausible differential expression (Figure 5B). Globally, the exposure of *B. uniformis* CECT 7771 to gum arabic and mucin appears to have a deep impact generating more transcriptional activity across its genome conversely to what observed in the response to WBE and inulin, which trend to trigger more concrete expression signals confined to a few and well-delimited gene clusters (Figure S1).

#### Gum arabic

The exploratory gene expression pattern derived from gum arabic fermentation suggests that 220 genes are significantly up-regulated whereas only 30 are down-regulated. The functional annotation of the respective set of genes, using the KEGG Modules, indicates that gum arabic triggers a higher expression of genes involved in central metabolic functions such as reductive pentose phosphate and citrate cycles (M00167 and M00173, respectively). Additionally, gum arabic induces over-expression of a wide variety of genes involved in starch and sucrose metabolism (ko:00500 pathway) like endoglucanases (K01179), glucosidases (K01182, K05349), and debranching enzymes (K01196). Notwithstanding, other glycan metabolic pathways (e.g., galactose—ko:00052, fructose and mannose—ko:00051, and other glycan degradation—ko:00511) also seem to be up-regulated by the significantly higher transcriptional signal detected for galactosidase (K01190, K12111), fucosidase (K15923), epimerase (K01785), mannosidase (K01218), isomerase (K01813), arabinofuranosidase (K01209), and epimerase (K01785) genes. Importantly, we disclosed a potential over-expression of the phosphate butyryltransferase (K00634) and butyrate kinase (K00929) genes, involved in production of the short-chain fatty acid (SCFA) butyrate. As a consequence of the great number and variety of genes activated and involved in carbohydrate catabolic and anabolic pathways, a wide range of amino acid metabolism pathways were seen to be probably up-regulated as well, except for metabolic pathways involving sulfur-containing amino acids (e.g., cysteine and methionine) which appear to be down-regulated. Globally, it seems that sulfur relay system is weakened with gum arabic given that expression of other important enzymes for this process as the tRNA-uridine 2-sulfurtransferase (K00566) and cysteine synthase A (K01738) are attenuated as well. This results could partially explain the growth phenotype of *B. uniformis* CECT 7771 in gum arabic given that trafficking and delivering of sulfur is indispensable for biosynthesis of Fe/S clusters in proteins, enzyme cofactors, and tRNAs (Mueller, 2006). The ample range of functions disclosed to be boosted by gum arabic is consistent with its complex nature based on a mixture of very different branched polysaccharides. Notably, when the specific set of genes differentially expressed in gum arabic cultures (see Figure 5A, *N* = 13) was analyzed, from the functional point of view, we disclosed genes encoding proteins and enzymes highly related with rhamnose metabolism such as L-rhamnose-H+ transport protein (K02856), L-rhamnose isomerase (K01813), and rhamnulokinase (K00848). This result makes sense since the complex mixture of the gum arabic includes rhamnose (12–16%) (Ali et al., 2009) and this appears to be part of no other carbon sources employed in this study.

#### WBE

The expression pattern associated with WBE fermentation was the best defined in terms of the low number of clustered genes showing differential expression when compared to the glucose response (see Figure S1). As expected and given its composition, we observed trends of over-expression in genes associated with metabolism of arabinoxylans. Thus, we found that a higher number of DNA reads were mapped against xylulokinase (K00854), L-arabinose isomerase (K01804), xylose isomerase (K01805), alpha-N-arabinofuranosidase (K01209), beta-D-xylosidase (K15920), and arabinoxylanarabinofuranohydrolase (K15921) genes. By contrast, we detected that lysine biosynthesis (ko:M00527) could be attenuated by down-regulation of the diaminopimelate epimerase (K01778) and LL-diaminopimelate aminotransferase (K10206) genes. Moreover, biosynthesis of other amino acids may also be decreased given that glutamate (K00265, K00266), glutamine (K01915), and asparagine (K01953) synthases appeared to be down-regulated as well. Among the set of genes specifically associated with WBE (see Figure 5A, *N* = 10) we found an over-expression pattern for those encoding ABC transporter proteins (K02003, K02004, K2005), outer membrane protein (K012538), and evidently genes encoding enzymes for xylan metabolism as beta-glucosidase (K05349), xylose isomerase (K01805), and beta-D-xylosidase (K15920).

#### Inulin

Similarly to WBE, inulin induced differential expression in a low number of genes. The functional annotation of genes with a tendency to be up-regulated in response to inulin was scarce. In consequence, we could not distinguish any molecular functions particularly related to this carbon source, except for the orthologs K02004/K02003/K01990, and K01190 which encodes respective genes for ABC sugar transporters and beta-galactosidase proteins comprising general functions into the carbohydrate metabolic pathway. Interestingly, inulin also trends to limit the expression of genes related to glutamate (K00266), glutamine (K01915), and asparagine (K01953) biosynthesis as previously seen with WBE.

#### Pectin

Opposite to WBE and inulin, the use of pectin as carbon source significantly induced the transcription of several genes across the *B. uniformis* CECT 7771 genome. In sum, 118 genes showed a tendency to be up-regulated and 46 to be down-regulated. Over-expression signals from the 6-phosphofructokinase 1 (K00850), biphosphoglycerate mutase (K01834), and the fructose-bisphosphate aldolase (K11645) indicate that glycolysis and gluconeogenesis pathways could be more active in presence of pectin than glucose. Likewise, the more abundant expression of genes related to galactose metabolism (ko:00052) such as the beta-galactosidase (K01190), UDP-glucose 4-epimerase, and the same 6-phosphofructokinase 1 (K00850) fits with the primary composition of the pectin consisting of galacturonic acid polymers. Other genes involved in carbohydrate metabolism whose expression was induced by pectin include the ABC permeases and transporters (K01990, K02003, K02004), glycogen phosphorylase (K00688), alpha-N-arabinofuranosidase (K01209), UDP-N-acetylglucosamine 4,6-dehydratase (K15894), glycosyltransferase EpsJ (K19427), and the genes encoding enzymes involving in polysaccharide biosynthesis as the heptose III glucuronosyltransferase (K019354) and Fuc2NAc and GlcNAc transferase (K13007). Remarkably, pectin also seems to activate the butanoate metabolism (ko:00650) by over-expression of the phosphate butyryltransferase (K00634), butyrate kinase (K00929), and glutamate decarboxylase (K01580) genes involved in production of pivotal metabolites for human colon function as butyrate and GABA (Bourassa et al., 2016; Mazzoli and Pessione, 2016). The over-expression pattern observed for the glutamate:GABA antiporter (K20265) could be another indication that pectin does induce production and release of GABA to the extracellular media by *B. uniformis* CECT 7771. The genes encoding the glycosyltransferase EpsJ, Fuc2NAc, and GlcNAc transferase and the glutamate decarboxylase and glutamate:GABA antiporter appear to be specifically involved in the response to pectin fermentation by *B. uniformis* CECT 7771. On the other hand, signals for gene expression attenuation seem to affect predominantly the alanine, aspartate and glutamate (ko:00250) and nitrogen (ko:00910) metabolism as well as arginine (ko:00220) and lysine (ko:00300) biosynthesis by down-regulation of the glutamate synthase (NADPH/NADH) large chain (K00265), glutamate synthase (NADPH/NADH) small chain (K00266), aspartate-ammonia ligase (K01914), glutamine synthetase (K01915), asparagine synthase (K01953), diaminopimelate epimerase (K01778), and LL-diaminopimelate aminotransferase (K10206) genes.

#### Mucin

The expression patterns of the *B. uniformis* CECT 7771 genome as a consequence of mucin utilization comprised up-regulation and down-regulation of 423 and 115 genes, respectively. Most of the genes positively and negatively regulated by mucin appear to be specific to this carbon source as well as the metabolic pathways potentially altered. Mucin caused the most complex gene expression response of *B. uniformis* CECT 7771 when compared to gum arabic, WBE, inulin, and pectin. Functional categorization of genes with a trend to be over-expressed includes genes involved in glycolysis and galactose degradation as well as other genes involved in keratan sulfate, chondroitin sulfate, heparan sulfate, dermatan sulfate, D-galacturone, and D-glucuronate degradation. The genes globally up-regulated and involved in molecular pathways like the amino sugar and nucleotide sugar metabolism (ko:00520), the starch and sucrose metabolism (ko:00500), the galactose metabolism (ko:00052), the fructose and mannose metabolism (ko:00051), and the glycosaminoglycan degradation (ko:00531), and representing the so called PULs (Polysaccharide Utilization Loci) of *B. uniformis* CECT 7771 for mucin glycan degradation are listed in Table S4. Similarly to that observed in gum arabic and pectin cultures, the amino acid metabolism was connected with sugar degradation pathways, therefore, we also detected up-regulation of genes involved in glycine-serine-threonine (ko:00260), alanine-aspartate-glutamate (ko:00250), phenyalanine (ko:00360), tryptophan (ko:00380), and arginine (ko:00220) metabolism. Analogously to that found in gum arabic and pectin cultures, mucin glycans induced the highest increased expression signal for genes involved in butyrate production (K00929, K00634) (see Figure 5C). Outstandingly, the genes with a down-regulation pattern of expression indicate that biosynthesis of long-chain fatty acids is mainly affected (Figure 5C). Consequently, the genes involved in the biotin (K00652, K00833, K01935), acyl-CoA (K01897), and pimeloyl-ACP biosynthesis (K02169, K09789) as well as the *fabD* (K00645), *fabF* (K09458), and *fabI* (K00208) orthologs associated with the initiation and elongation of fatty acids were determined to have a lower expression level in mucin than glucose containing media. Additionally, lysine and branched-chain amino acids biosynthesis also seem to be reduced when mucin was used as carbon source. Globally, the nitrogen metabolism appears to be also constrained given the lower expression of genes related to glutamate-glutamine (K00262, K00265, K00266, K01915) and ornithine biosynthesis (K00145, K00821). Altogether, the expression signals observed indicate that multiple pathways involve carbohydrate degradation are activated in *B. uniformis* CECT 7771, likely due to the great variety of glycans attached to mucus proteins. Therefore, is plausible to assign a mucin degrader role to *B. uniformis* CECT 7771 that seems to be further supported by the up-regulation of the ortholog *clpB* encoding the ATP-dependent Clp protease (K03695).

### qPCR validation of differential gene expression

Despite the consistency among the metabolic functions up- and down-regulated depending on the carbon source used by *B. uniformis* CECT 7771 for growing, we validated the changes in expression of a set of 8 genes by qPCR, using replicated and non-pooled total RNA samples remaining from the exploratory whole-transcriptome experiment. These genes were selected for their possible role in the host physiology. In particular we analyzed the relative expression of following genes: BUNIF7771_0544 (K01580—glutamate decarboxylase), BUNIF7771_0548 (K20265—glutamate:GABA antiporter), BUNIF7771_1883 (K00929—butyrate kinase), BUNIF7771_0387 (K09789—pimeloyl-ACP methyl ester esterase), BUNIF7771_3732 (K01915—glutamine synthase), BUNIF7771_4131 (K00208—enoyl-ACP reductase I), BUNIF7771_1668 (K01813—L-rhamnose isomerase), and BUNIF7771_3473 (K01805—xylose isomerase). When we compared the respective and relative gene expression to that found in the presence of glucose, we confirmed almost all the gene expression patterns inferred from the transcriptome analysis (Figure S2).

### GABA production by *B. uniformis* CECT 7771

**Figure 6:**
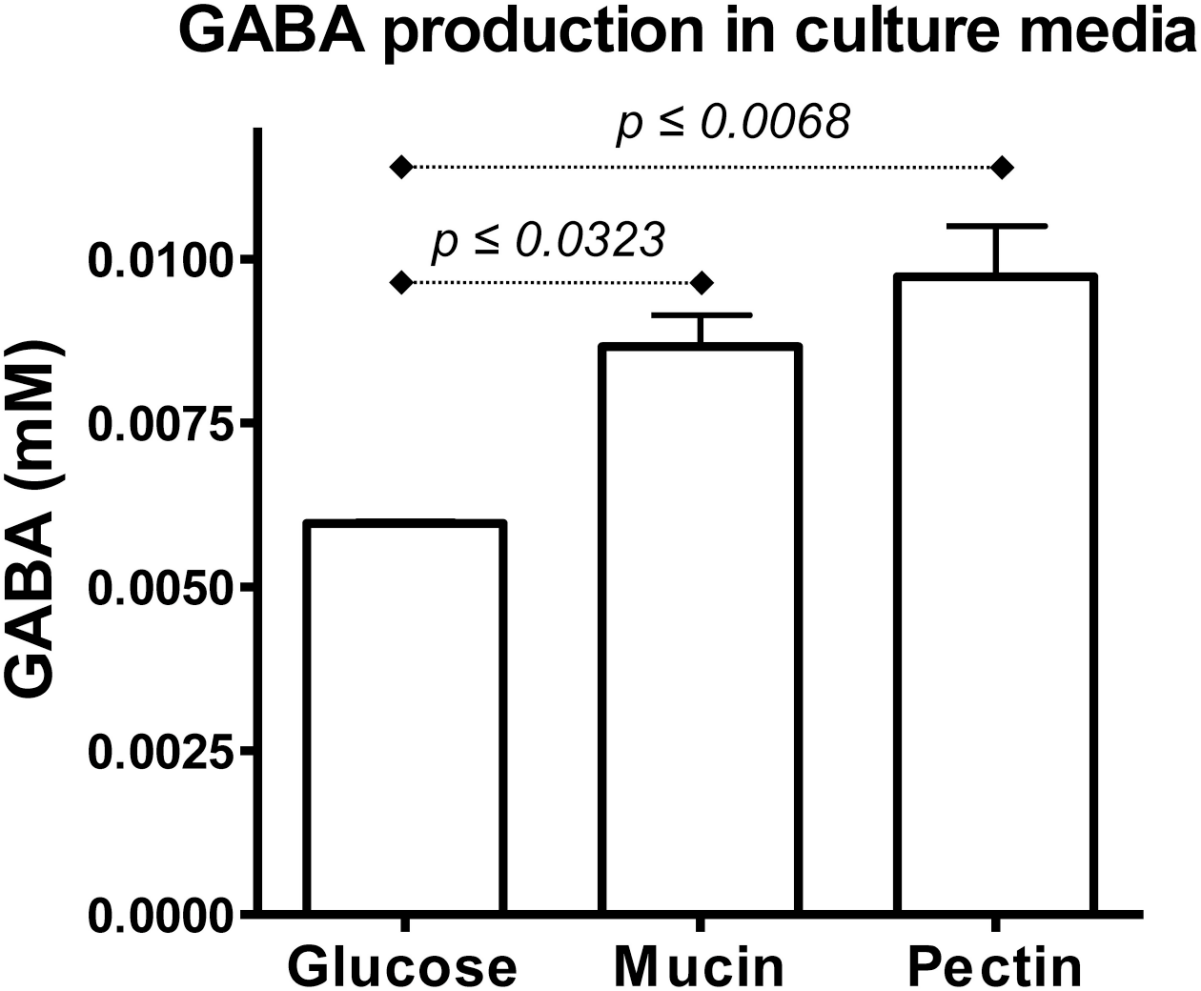
Quantification of GABA production in culture supernatants. Culture supernatants from conditions where over expression of glutamate decarboxylase (BUNIF7771_0544–K01580) and glutamate:GABA antiporter (BUNIF7771_0548–K20265) genes were inferred from the exploratory RNAseq and corroborated by qPCR were subject to LC-MS analysis in order to determine the GABA concentration at log phase of respective cultures (OD_600_ ~ 0.4). The comparisons were made using three independent replicates per condition and statistical analysis was assessed through pairwise *t-*test with Bonferroni correction.

Once we corroborated the expression pattern observed for *B. uniformis* CECT 7771 genes probably involved in GABA production by qPCR, we further analyzed the GABA concentration in supernatants of cultures supplemented with mucin and pectin that showed a clear over-expression of glutamate decarboxylase (BUNIF7771_0544) and glutamate:GABA antiporter (BUNIF7771_0544) genes (Figure S2). We observed that mucin and pectin increased the GABA concentration in the extracellular media by 45 and 63%, respectively, compared to glucose (Figure 6). These results together with the over-expression pattern inferred from the RNAseq approach, definitively confirm the role of different carbon sources in production of bioactive derived metabolites other than SCFAs, and, on the basis, suggest that innovative synbiotic products combining *B. uniformis* CECT 7771 with pectin and/or glycans similar to those covalently attached to mucins could be developed and functionally tested in pre-clinical trials.

## Conclusions

Using nanopore-based technology we could obtain reliable data to assembly a draft genome of the strain *B. uniformis* CECT 7771. The assembly and the primary sequence of the superscaffolds obtained were refined with paired-end transcriptome data permitting the prediction of more than 5,200 ORFs to be encoded in this human symbiont strain. Although genome information of other *B. uniformis* strains are publicly available in different biological databases, we presented for the first time the comparative analysis of such genomes describing their glycolytic capability. Our results indicate that *B. uniformis* strains have the largest repertoire of CAZy genes among *Bacteroides* species, and this set of genes outnumbers those observed in the archetype species *B. tethaiotaomicron* and the highly versatile species *B. cellulosyliticus*. To proof the glycan degradation versatility inferred *in silico* from the *B. uniformis* CECT 7771 genome we demonstrated that it was able to use all carbon sources tested but with different ability. Similarly, the genome response to the different glycans used induced different expression patterns with characteristic metabolic outputs. Our data indicates that *B. uniformis* CECT 7771 is able to utilize the *O*-glycans covalently attached to mammal mucin proteins as well as previously reported for *B. tethaiotaomicron* (Sonnenburg et al., 2005) and *B. fragilis* (Roberton and Stanley, 1982). Consequently, it is expected that *B. uniformis* is a mucin-degrader bacteria and that predominantly colonizes the mucosal surface and, therefore, be tightly interacting with the host. Our data also indicate that the wide repertoire of glycans being *O*-linked to mucus layer proteins at the intestinal epithelium could be enough to promote the growth and persistency of *B. uniformis* in absence of dietary fiber. The fact that mucin *O-*glycans induce a higher production of butyrate in the growth medium by up-regulating the genes responsible for its production in *B. uniformis* CECT 7771 clearly disclose a metabolic circuit, which could be the cornerstone of a mutualistic relationship between the bacterium and the host, given that butyrate reciprocally induces production of mucin in colorectal cells (Jung et al., 2015). Remarkably, our results indicate that degradation of mucin *O-*glycans by *B. uniformis* CECT 7771 reduces the production of long-chain fatty acids and expression of the acyl carrier protein (ACP), fundamental components for the LPS (lipopolysaccharide) biosynthesis in Gram-negative bacteria (Masoudi et al., 2014; Emiola et al., 2015). Accordingly, we hypothesized this could have a positive effect on gut health given the recognized role of LPS endotoxin in the chronic low-grade inflammation underlying obesity (Cani et al., 2007). Our study also shows that the use of pectin by *B. uniformis* CECT 7771 enhances the butanoate metabolism, therefore increasing the production of butyrate and specially the production of the inhibitory neurotransmitter GABA, which could respectively contribute to strengthen the gut barrier and hypothetically play a role in mental health from the intestine (Dinan and Cryan, 2017; Sandhu et al., 2017).

Globally, our results shed light on the molecular basis of mutualism between the humans and an important component of the gut microbiota, such as *Bacteroides uniformis*, and of its dependency on the carbon source available as a nutrient for this bacterium. The ample repertoire of glycolytic activities observed in this mucin-degrader bacteria could have been the result of the need to consume primarily endogenous nutrients to survive in the infant's gut, which seem to exert a deep impact on *B. uniformis* metabolism, even when dietary glycans are absent. This could also explain the presence of this bacterial species in the gut of exclusively breast-fed infants before the introduction of solid food, including plant oligo- and polysasccharides. Nevertheless, the results are only based on an *in vitro* study using single strain cultures and the possibility that the global response of *B. uniformis* CECT 7771 observed in this study could be modified to some extent by the *in vivo* multiple competitive and cooperative interactions with other microbial species in the gut should not be disregarded (Mitri and Foster, 2013). Future *in vivo* gene expression and metabolome analyses should be warranted to progress in the understanding of the metabolic response of *B. uniformis* inside the host and the possible physiological effects.

## Author contributions

EGdP performed the cell cultures, isolation of nuclei acids, and qPCR experiments. ABP sequenced, assembled, and annotated the bacterial genome as well as performed transcriptome analysis. ABP and YS designed and directed the study. All authors contributed to manuscript writing.

### Conflict of interest statement

The authors declare that the research was conducted in the absence of any commercial or financial relationships that could be construed as a potential conflict of interest.

## Supplementary material

Figure S1 Expression patterns across the *B. uniformis* CECT 7771 genome. GFOLD scores obtained for every single gene predicted to be encoded by the *B. uniformis* CECT 7771 genome were plotted for all culture conditions tested in this study. The dashed lines indicated the threshold for up- (≥1.5) or down-regulation (≤ −1.5). The more distant GFOLD values are from zero, the darker the dots become.

Figure S2 Gene expression assessment by qPCR. A set of 8 different genes with differential expression patterns in different culture conditions were evaluated to corroborate the RNAseq analysis. In this case, non-pooled RNA samples were used, the expression of 16S was used as normalizer (endogenous control), and the glucose sample was used as the reference. Eight panels are presented with respective expression data in terms of RQ (2^−ΔΔCt^) for all genes selected. The respective gene tag (BUNIF7771_XXXX) and the gene functional annotation according to KEGG Orthology are showed as headers. ^*^p < 0.05.

Table S1 Primers for qPCR analysis.

Table S2 Average Nucleotide Identity (ANI) analysis among different strains of *B. uniformis*.

Table S3 Strain-specific CAZy functions in *B. uniformis*.

Table S4 Functional analysis of sugar metabolism genes over-expressed in mucin-containing media.

## Abbreviations

ACP: Acyl carrier protein
ARDB: Antibiotic Resistance Genes Database
ATCC: American Type Culture Collection
AXOS: arabinoxylan-oligosaccharides
CAT: Cazymes Analysis Toolkit server
CAZy: Carbohydrate Enzyme Database
cDNA: complementary DNA
CECT: Spanish Type Culture Collection
CRISPR: clustered regularly interspaced short palindromic repeats
dsDNA: double-stranded DNA
ENA: European Nucleotide Archive
GABA: gamma-aminobutyric acid
GH: glycoside hydrolase
GIT: human gastrointestinal tract
GT: glycosyl transferase
KEGG: Kyoto Encyclopedia of Genes and Genomes
LC: liquid chromatography
LPS: lipopolysaccharide
Mbp: mega base pair
MS: mass spectrometry
MvirDB: Microbial database of protein toxins,virulence factors and antibiotic resistance genes for bio-defence applications
NTCT: National Collection of Type Cultures
OD_600_: optical density at 600 nm
ORF: open reading frame
PUL: polysaccharide utilization loci
qPCR: quantitative polymerase chain reaction
RPKM: reads per kilobase of transcript per million mapped reads
RQ: relative quantification
RSMD: root-mean-square deviation
SMART: Simple Modular Architecture Research Tool
SR: specificity regions
ssDNA: single-stranded DNA
UPGMA: unweighted pair group method with arithmetic mean
VFDB: Virulence Factors Database
WBE: wheat bran extract
WGS: whole genome sequence.
