# Supplementary figures and images for: The Glycolytic Versatility of Bacteroides uniformis CECT 7771 and Its Genome Response to Oligo and Polysaccharides
Source: Front Cell Infect Microbiol. 2017 Aug 25;7:383. doi: 10.3389/fcimb.2017.00383 (PMC5609589; doi:10.3389/fcimb.2017.00383)

Gum arabic

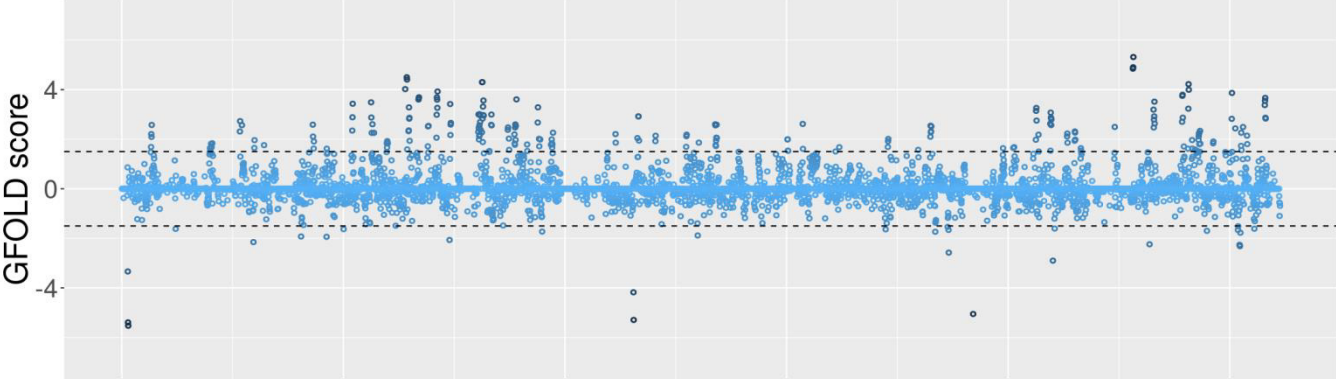

WBE

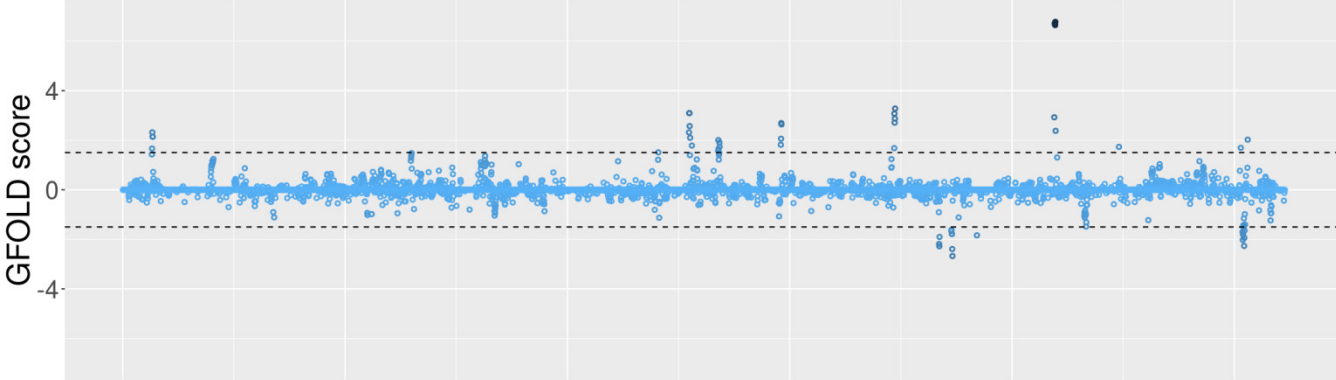

Inulin

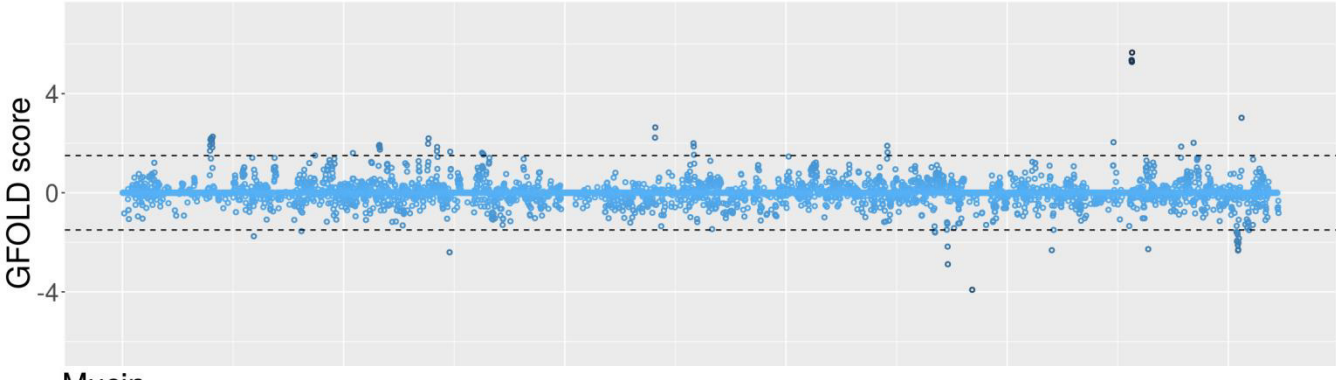

Mucin

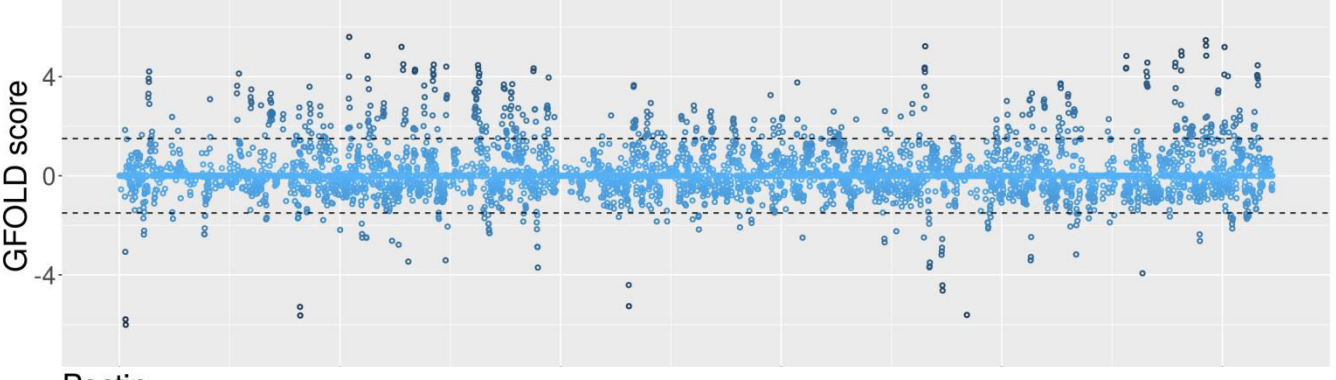

Pectin

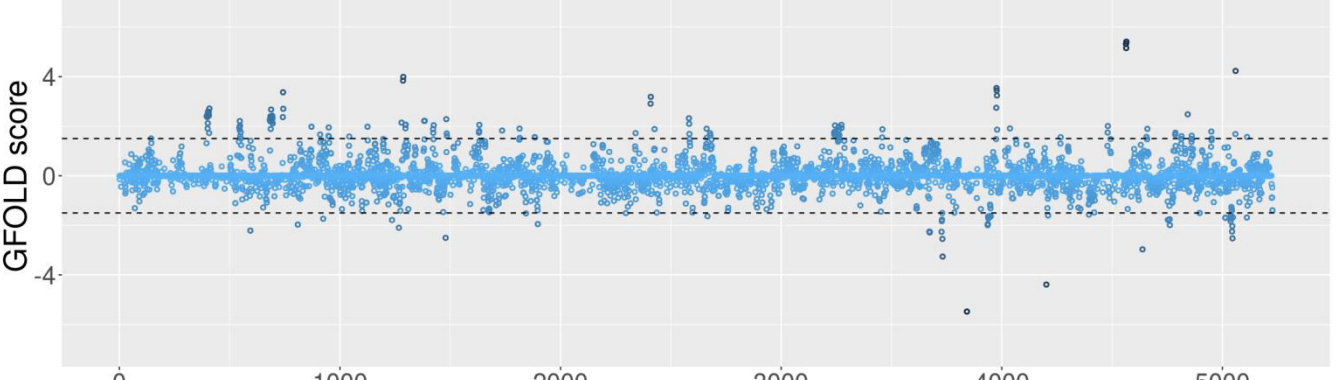

Genes

Supplement: Figure S1 — Expression patterns across the B. uniformis CECT 7771 genome. GFOLD scores obtained for every single gene predicted to be encoded by the B. uniformis CECT 7771 genome were plotted for all culture conditions tested in this study. The dashed lines indicated the threshold for up- (≥1.5) or down-regulation (≤ −1.5). The more distant GFOLD values are from zero, the darker the dots become. [file Image1.PDF]
