# Supplementary material for: The Glycolytic Versatility of Bacteroides uniformis CECT 7771 and Its Genome Response to Oligo and Polysaccharides
Source: Front Cell Infect Microbiol. 2017 Aug 25;7:383. doi: 10.3389/fcimb.2017.00383 (PMC5609589; doi:10.3389/fcimb.2017.00383)
Supplement: Figure S2 — Gene expression assessment by qPCR. A set of 8 different genes with differential expression patterns in different culture conditions were evaluated to corroborate the RNAseq analysis. In this case, non-pooled RNA samples were used, the expression of 16S was used as normalizer (endogenous control), and the glucose sample was used as the reference. Eight panels are presented with respective expression data in terms of RQ (2−ΔΔCt) for all genes selected. The respective gene tag (BUNIF7771_XXXX) and the gene functional annotation according to KEGG Orthology are showed as headers. *p < 0.05. [file Image2.PDF]

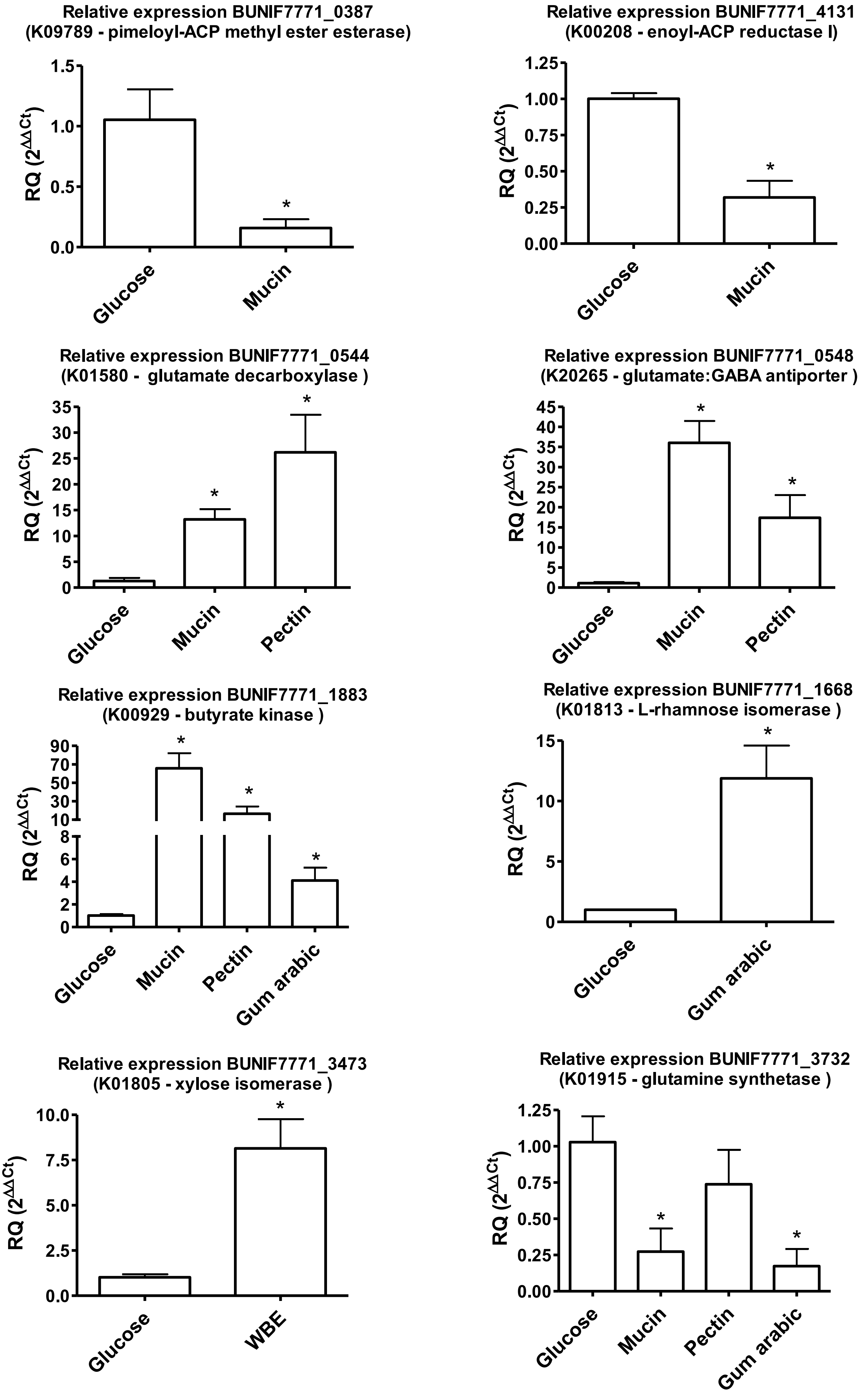
Relative expression BUNIF7771_0387
(K09789 - pimeloyl-ACP methyl ester esterase)
RQ ($2^{\Delta\Delta Ct}$)
0.0
0.5
1.0
1.5
*
Glucose
Mucin
Relative expression BUNIF7771_4131
(K00208 - enoyl-ACP reductase I)
RQ ($2^{\Delta\Delta Ct}$)
0.00
0.25
0.50
0.75
1.00
1.25
*
Glucose
Mucin
Relative expression BUNIF7771_0544
(K01580 - glutamate decarboxylase)
RQ ($2^{\Delta\Delta Ct}$)
0
5
10
15
20
25
30
35
*
*
Glucose
Mucin
Pectin
Relative expression BUNIF7771_0548
(K20265 - glutamate:GABA antiporter)
RQ ($2^{\Delta\Delta Ct}$)
0
5
10
15
20
25
30
35
40
45
*
*
Glucose
Mucin
Pectin
Relative expression BUNIF7771_1883
(K00929 - butyrate kinase)
RQ ($2^{\Delta\Delta Ct}$)
0
2
4
6
8
10
30
50
70
90
*
*
*
Glucose
Mucin
Pectin
Gum arabic
Relative expression BUNIF7771_1668
(K01813 - L-rhamnose isomerase)
RQ ($2^{\Delta\Delta Ct}$)
0
5
10
15
*
Glucose
Gum arabic
Relative expression BUNIF7771_3473
(K01805 - xylose isomerase)
RQ ($2^{\Delta\Delta Ct}$)
0.0
2.5
5.0
7.5
10.0
*
Glucose
WBE
Relative expression BUNIF7771_3732
(K01915 - glutamine synthetase)
RQ ($2^{\Delta\Delta Ct}$)
0.00
0.25
0.50
0.75
1.00
1.25
*
*
Glucose
Mucin
Pectin
Gum arabic
