# Supplementary material for: The Glycolytic Versatility of Bacteroides uniformis CECT 7771 and Its Genome Response to Oligo and Polysaccharides
Source: Front Cell Infect Microbiol. 2017 Aug 25;7:383. doi: 10.3389/fcimb.2017.00383 (PMC5609589; doi:10.3389/fcimb.2017.00383)
Supplement: Table S1 — Primers for qPCR analysis. [file Table1.DOCX]

Table S1. Primers for qPCR analysis.

| **Primer name** | **Primer sequence 5'-3'** | **PCR product** |
| --- | --- | --- |
| BU-387F | GGAATTCCCGAAAAGACCTTCCG |  |
| BU-387R | GTAGGCATTGCTGTCTTTGCACAT | 99bp |
| BU-544F | TATGCAACCAAGCTGATGAACGAAG |  |
| BU-544R | AGAGGTTGGCCACGATGTTGATAC | 112bp |
| BU-548F | TGGATTTCCACAGCAGCTTCTTCC |  |
| BU-548R | TCTTTTACGTGGATACCGCCCATT | 115bp |
| BU-1668F | AACATTTCGAAAGCTGGATGCAATGG |  |
| BU-1668R | GTCCGGATGAGCCAGAGAAAGAT | 113bp |
| BU-1883F | GGAGGCCAATGACATTCCCTTTAAG |  |
| BU-1883R | AGCATATCGTCCAACATGGCATCG | 114bp |
| BU-3473F | CGAAGTGAACCACGCTACTTTGG |  |
| BU-3473R | GTTCTGGTAGTCACCACGGTTG | 112bp |
| BU-3732F | ATGTTCGAGTCCATCGGTGTCATG |  |
| BU-3732R | AGCACGCGGGCTTCAATCTGTATT | 104bp |
| BU-4131F | GGTGCTCTGAATGAGCAGTCCAT |  |
| BU-4131R | CATACGGACAGCAACCGGTGTAT | 102bp |
| pBU7771F | GCCCCCTTAACCCCCTGTCA |  |
| pBU7771R | CACTCCCCGGCTTCACTGCT | 111bp |
| 16S-F | CACGGGTGAGTAACACGTATCC |  |
| 16S-R | GCATCCCCATCGATAACCGAAA | 132bp |
