# Supplementary material for: The Glycolytic Versatility of Bacteroides uniformis CECT 7771 and Its Genome Response to Oligo and Polysaccharides
Source: Front Cell Infect Microbiol. 2017 Aug 25;7:383. doi: 10.3389/fcimb.2017.00383 (PMC5609589; doi:10.3389/fcimb.2017.00383)
Supplement: Table S2 — Average Nucleotide Identity (ANI) analysis among different strains of B. uniformis. [file Table2.DOCX]

Table S2. Average Nucleotide Identity (ANI) analysis among different strains of *B. uniformis*.

|  | ***B. uniformis***  **CECT 7771** | ***B. uniformis***  **ATCC 8492** | ***B. uniformis* CL03T00C23** | ***B. uniformis***  **3978-T3i** | ***B. uniformis* dnLKV2** | ***B. stercoris***  **ATCC 43183** | ***B. caccae***  **ATCC 43185** | ***B. fragilis***  **NCTC 9343** | ***B. vulgatus***  **ATCC 8482** | ***B. thetaiotaomicron* VPI 5482** |
| --- | --- | --- | --- | --- | --- | --- | --- | --- | --- | --- |
| ***B. uniformis***  **CECT 7771** | 100  100 |  |  |  |  |  |  |  |  |  |
| ***B. uniformis***  **ATCC 8492** | 99.6  98.4 | 100  100 |  |  |  |  |  |  |  |  |
| ***B. uniformis* CL03T00C23** | 99.7  98.1 | 99.9  98.4 | 100  100 |  |  |  |  |  |  |  |
| ***B. uniformis***  **3978-T3i** | 99.7  98.2 | 99.9  98.5 | 99.9  98.4 | 100  100 |  |  |  |  |  |  |
| ***B. uniformis* dnLKV2** | 99.6  97.3 | 99.9  97.7 | 99.9  98.0 | 99.9  97.9 | 100  100 |  |  |  |  |  |
| ***B. stercoris***  **ATCC 43183** | 95.3  86.8 | 94.4  86.7 | 95.2  85.4 | 94.9  86.5 | 94.7  85.5 | 100  100 |  |  |  |  |
| ***B. caccae***  **ATCC 43185** | 85.5  84.1 | 85.3  85.1 | 86.0  83.5 | 85.8  83.4 | 85.8  84.2 | 90.1  84.4 | 100  100 |  |  |  |
| ***B. fragilis***  **NCTC 9343** | 83.3  84.3 | 83.3  86.2 | 84.2  84.0 | 83.9  84.3 | 83.8  83.8 | 87.2  84.5 | 93.8  83.9 | 100  100 |  |  |
| ***B. vulgatus***  **ATCC 8482** | 91.1  92.4 | 90.6  93.7 | 91.3  90.9 | 91.0  92.5 | 91.1  87.4 | 92.9  92.4 | 94.4  88.1 | 93.0  85.7 | 100  100 |  |
| ***B. thetaiotaomicron* VPI 5482** | 88.1  88.3 | 87.9  87.3 | 88.5  86.4 | 88.3  86.7 | 88.2  85.6 | 90.7  88.2 | 96.7  85.12 | 96.5  84.3 | 95.8  88.6 | 100  100 |

Genome-wide comparisons among *B. uniformis* genomes and reference *B. stercoris, B. fragilis, B. caccae, B. vulgatus,* and *B. thetaiotaomicron* genomes. Two different comparisons were made using tetranucleotide distribution correlation (top numbers) and average nucleotide identity (ANI) based on MUMmer algorithm (bottom numbers) (Richter and Rossello-Mora, 2009).
