# Supplementary material for: The Glycolytic Versatility of Bacteroides uniformis CECT 7771 and Its Genome Response to Oligo and Polysaccharides
Source: Front Cell Infect Microbiol. 2017 Aug 25;7:383. doi: 10.3389/fcimb.2017.00383 (PMC5609589; doi:10.3389/fcimb.2017.00383)
Supplement: Table S3 — Strain-specific CAZy functions in B. uniformis. [file Table3.DOCX]

Table S3. Strain-specific CAZy functions in *B. uniformis*

| Strain | CAZy family | Function^1^ | EC number^1^ |
| --- | --- | --- | --- |
| *B. uniformis* CECT 7771 | GT64 | heparan α-N-acetylhexosaminyltransferase | 2.4.1.224 |
| *B. uniformis* ATCC 8492 | CE12 | pectin acetylesterase | 3.1.1.- |
|  | GH81 | endo-β-1,3-glucanase | 3.2.1.39 |
|  | GH115 | xylan α-1,2-glucuronidase | 3.2.1.131 |
|  | CBM67 | L-rhamnose binding activity | NA |
| *B. uniformis* CL03T00C23 | AA5 | galactose oxidase | 1.1.3.9 |
|  | GH123 | β-N-acetylgalactosaminidase | 3.2.1.53 |
|  | GH10 | endo-1,4-β-xylanase | 3.2.1.8 |
|  | GH67 | xylan α-1,2-glucuronidase | 3.2.1.131 |
|  | CBM3 | cellulose-binding activity | NA |
|  | CE6 | acetyl xylan esterase | 3.1.1.72 |
|  | CBM26 | starch-binding activity | NA |
|  | PL1 | pectate lyase | 4.2.2.2 |
| *B. uniformis* dnLKV2 | GH106 | α-L-rhamnosidase | 3.2.1.40 |
| *B. uniformis* 3978-T3i | GT31 | N-acetyllactosaminide β-1,3-N-acetylglucosaminyltransferase | 2.4.1.149 |
|  | GH4 | α-glucosidase  α-galactosidase | 3.2.1.20  3.2.1.22 |
|  | GT56 | lipid II Fuc4NAc transferase | 2.4.1.- |
|  | CE2 | acetyl xylan esterase | 3.1.1.72 |
|  | GT25 | β-1,3-glucosyltransferase  β-1,2-glucosyltransferase  β-1,2-galactosyltransferase  β-1,4-galactosyltransferase | 2.4.1.- |

1 The function annotation and Enzyme Classification (EC) numbers were obtained from respective family information at CAZy database.
