# Supplementary material for: The Glycolytic Versatility of Bacteroides uniformis CECT 7771 and Its Genome Response to Oligo and Polysaccharides
Source: Front Cell Infect Microbiol. 2017 Aug 25;7:383. doi: 10.3389/fcimb.2017.00383 (PMC5609589; doi:10.3389/fcimb.2017.00383)
Supplement: Table S4 — Functional analysis of sugar metabolism genes over-expressed in mucin-containing media. [file Table4.DOCX]

Table S4. Functional analysis of sugar metabolism genes over-expressed in mucin-containing media

| Gene tag | KEGG id | GFOLD score | Functional annotation |
| --- | --- | --- | --- |
| BUNIF7771_0102 | K00616 | 1.65 | E2.2.1.2, talA, talB; transaldolase [EC:2.2.1.2] |
| BUNIF7771_0131 | K00700 | 3.15 | GBE1, glgB; 1,4-alpha-glucan branching enzyme [EC:2.4.1.18] |
| BUNIF7771_0132 | K01187 | 3.31 | malZ; alpha-glucosidase [EC:3.2.1.20] |
| BUNIF7771_0411 | K06158 | 3.09 | ABCF3; ATP-binding cassette, subfamily F, member 3 |
| BUNIF7771_0412 | K06158 | 1.57 | ABCF3; ATP-binding cassette, subfamily F, member 3 |
| BUNIF7771_0595 | K02039 | 3.17 | phoU; phosphate transport system protein |
| BUNIF7771_0596 | K02036 | 2.73 | pstB; phosphate transport system ATP-binding protein [EC:3.6.3.27] |
| BUNIF7771_0597 | K02038 | 3.01 | pstA; phosphate transport system permease protein |
| BUNIF7771_0598 | K02037 | 3.49 | pstC; phosphate transport system permease protein |
| BUNIF7771_0599 | K02040 | 2.94 | pstS; phosphate transport system substrate-binding protein |
| BUNIF7771_0685 | K11645 | 3.05 | fbaB; fructose-bisphosphate aldolase, class I [EC:4.1.2.13] |
| BUNIF7771_0689 | K00850 | 2.59 | pfkA, PFK; 6-phosphofructokinase 1 [EC:2.7.1.11] |
| BUNIF7771_0690 | K03455 | 2.42 | TC.KEF; monovalent cation:H+ antiporter-2, CPA2 family |
| BUNIF7771_0692 | K00688 | 2.25 | PYG, glgP; glycogen phosphorylase [EC:2.4.1.1] |
| BUNIF7771_0693 | K00688 | 2.48 | PYG, glgP; glycogen phosphorylase [EC:2.4.1.1] |
| BUNIF7771_0871 | K01990 | 1.71 | ABC-2.A; ABC-2 type transport system ATP-binding protein |
| BUNIF7771_1128 | K16147 | 2.51 | glgE; starch synthase (maltosyl-transferring) [EC:2.4.99.16] |
| BUNIF7771_1138 | K01183 | 2.83 | E3.2.1.14; chitinase [EC:3.2.1.14] |
| BUNIF7771_1139 | K02429 | 1.82 | fucP; MFS transporter, FHS family, L-fucose permease |
| BUNIF7771_1333 | K12373 | 1.89 | HEXA_B; hexosaminidase [EC:3.2.1.52] |
| BUNIF7771_1342 | K00845 | 1.86 | glk; glucokinase [EC:2.7.1.2] |
| BUNIF7771_1379 | K05349 | 2.00 | bglX; beta-glucosidase [EC:3.2.1.21] |
| BUNIF7771_1483 | K01201 | 3.11 | GBA, srfJ; glucosylceramidase [EC:3.2.1.45] |
| BUNIF7771_1610 | K01787 | 3.38 | RENBP; N-acylglucosamine 2-epimerase [EC:5.1.3.8] |
| BUNIF7771_1611 | K16139 | 2.68 | uidB, gusB; glucuronide carrier protein |
| BUNIF7771_1612 | K03292 | 2.48 | TC.GPH; glycoside/pentoside/hexuronide:cation symporter, GPH family |
| BUNIF7771_1614 | K01218 | 2.79 | gmuG; mannan endo-1,4-beta-mannosidase [EC:3.2.1.78] |
| BUNIF7771_1628 | K01218 | 3.39 | gmuG; mannan endo-1,4-beta-mannosidase [EC:3.2.1.78] |
| BUNIF7771_1634 | K01179 | 2.12 | E3.2.1.4; endoglucanase [EC:3.2.1.4] |
| BUNIF7771_1779 | K00864 | 2.09 | glpK, GK; glycerol kinase [EC:2.7.1.30] |
| BUNIF7771_1781 | K00615 | 3.70 | E2.2.1.1, tktA, tktB; transketolase [EC:2.2.1.1] |
| BUNIF7771_1798 | K01190 | 1.80 | lacZ; beta-galactosidase [EC:3.2.1.23] |
| BUNIF7771_1799 | K01206 | 1.91 | FUCA; alpha-L-fucosidase [EC:3.2.1.51] |
| BUNIF7771_1882 | K03931 | 1.55 | ygjK; putative isomerase |
| BUNIF7771_1944 | K01132 | 2.52 | GALNS; N-acetylgalactosamine-6-sulfatase [EC:3.1.6.4] |
| BUNIF7771_1945 | K01195 | 1.70 | uidA, GUSB; beta-glucuronidase [EC:3.2.1.31] |
| BUNIF7771_1946 | K05349 | 3.96 | bglX; beta-glucosidase [EC:3.2.1.21] |
| BUNIF7771_2331 | K01179 | 3.59 | E3.2.1.4; endoglucanase [EC:3.2.1.4] |
| BUNIF7771_2572 | K01208 | 2.17 | cd, ma, nplT; cyclomaltodextrinase / maltogenic alpha-amylase / neopullulanase [EC:3.2.1.54 3.2.1.133 |
| BUNIF7771_2678 | K01209 | 2.42 | abfA; alpha-N-arabinofuranosidase [EC:3.2.1.55] |
| BUNIF7771_2685 | K01785 | 1.86 | galM, GALM; aldose 1-epimerase [EC:5.1.3.3] |
| BUNIF7771_2686 | K00849 | 1.76 | galK; galactokinase [EC:2.7.1.6] |
| BUNIF7771_2687 | K00849 | 1.78 | galK; galactokinase [EC:2.7.1.6] |
| BUNIF7771_3073 | K01960 | 3.76 | pycB; pyruvate carboxylase subunit B [EC:6.4.1.1] |
| BUNIF7771_3242 | K15894 | 1.51 | pseB; UDP-N-acetylglucosamine 4,6-dehydratase [EC:4.2.1.115] |
| BUNIF7771_3244 | K15897 | 1.76 | pseG; UDP-2,4-diacetamido-2,4,6-trideoxy-beta-L-altropyranose hydrolase [EC:3.6.1.57] |
| BUNIF7771_3263 | K19354 | 1.55 | waaH; heptose III glucuronosyltransferase [EC:2.4.1.-] |
| BUNIF7771_3273 | K01784 | 1.96 | galE, GALE; UDP-glucose 4-epimerase [EC:5.1.3.2] |
| BUNIF7771_3471 | K00854 | 1.85 | xylB, XYLB; xylulokinase [EC:2.7.1.17] |
| BUNIF7771_3474 | K08138 | 1.86 | xylE; MFS transporter, SP family, xylose:H+ symportor |
| BUNIF7771_3649 | K05546 | 3.58 | GANAB; alpha 1,3-glucosidase [EC:3.2.1.84] |
| BUNIF7771_3781 | K00874 | 1.53 | kdgK; 2-dehydro-3-deoxygluconokinase [EC:2.7.1.45] |
| BUNIF7771_4139 | K00845 | 1.98 | glk; glucokinase [EC:2.7.1.2] |
| BUNIF7771_4190 | K01179 | 2.99 | E3.2.1.4; endoglucanase [EC:3.2.1.4] |
| BUNIF7771_4195 | K05349 | 2.80 | bglX; beta-glucosidase [EC:3.2.1.21] |
| BUNIF7771_4258 | K06859 | 1.98 | pgi1; glucose-6-phosphate isomerase, archaeal [EC:5.3.1.9] |
| BUNIF7771_4260 | K01809 | 2.34 | manA, MPI; mannose-6-phosphate isomerase [EC:5.3.1.8] |
| BUNIF7771_4301 | K01196 | 3.29 | AGL; glycogen debranching enzyme [EC:2.4.1.25 3.2.1.33] |
| BUNIF7771_4303 | K07405 | 2.54 | E3.2.1.1A; alpha-amylase [EC:3.2.1.1] |
| BUNIF7771_4785 | K15923 | 2.97 | AXY8, FUC95A, afcA; alpha-L-fucosidase 2 [EC:3.2.1.51] |
| BUNIF7771_4786 | K05349 | 4.40 | bglX; beta-glucosidase [EC:3.2.1.21] |
| BUNIF7771_4787 | K01190 | 4.56 | lacZ; beta-galactosidase [EC:3.2.1.23] |
| BUNIF7771_4788 | K01811 | 3.54 | xylS, yicI; alpha-D-xyloside xylohydrolase [EC:3.2.1.177] |
| BUNIF7771_4924 | K01134 | 2.34 | ARSA; arylsulfatase A [EC:3.1.6.8] |
| BUNIF7771_5029 | K15921 | 1.75 | xynD; arabinoxylan arabinofuranohydrolase [EC:3.2.1.55] |
| BUNIF7771_5163 | K03332 | 1.54 | fruA; fructan beta-fructosidase [EC:3.2.1.80] |
